# Unraveling Regulation of the Small Heat Shock Proteins by the Heat Shock Factor *HvHsfB2c* in Barley: Its Implications in Drought Stress Response and Seed Development

**DOI:** 10.1371/journal.pone.0089125

**Published:** 2014-03-04

**Authors:** Palakolanu Sudhakar Reddy, Polavarapu B. Kavi Kishor, Christiane Seiler, Markus Kuhlmann, Lennart Eschen-Lippold, Justin Lee, Malireddy K. Reddy, Nese Sreenivasulu

**Affiliations:** 1 Leibniz Institute of Plant Genetics and Crop Plant Research (IPK), Gatersleben, Germany; 2 Department of Genetics, Osmania University, Hyderabad, India; 3 Research Group Abiotic Stress Genomics, Interdisciplinary Center for Crop Plant Research, Halle, Germany; 4 Leibniz Institute of Plant Biochemistry (IPB), Halle, Germany; 5 International Centre for Genetic Engineering and Biotechnology, New Delhi, India; University of Pecs Medical School, Hungary

## Abstract

The rapid increase in heat shock proteins upon exposure to damaging stresses and during plant development related to desiccation events reveal their dual importance in plant development and stress tolerance. Genome-wide sequence survey identified 20 non-redundant small heat shock proteins (*sHsp*) and 22 heat shock factor (*Hsf*) genes in barley. While all three major classes (A, B, C) of Hsfs are localized in nucleus, the 20 sHsp gene family members are localized in different cell organelles like cytoplasm, mitochondria, plastid and peroxisomes. *Hsf* and *sHsp* members are differentially regulated during drought and at different seed developmental stages suggesting the importance of chaperone role under drought as well as seed development. *In silico cis*-regulatory motif analysis of *Hsf* promoters showed an enrichment with abscisic acid responsive *cis*-elements (ABRE), implying regulatory role of ABA in mediating transcriptional response of *Hv*s*Hsf* genes. Gene regulatory network analysis identified *HvHsfB2c* as potential central regulator of the seed-specific expression of several *HvsHsps* including *17.5CI sHsp*. These results indicate that *HvHsfB2c* is co-expressed in the central hub of small Hsps and therefore it may be regulating the expression of several *HvsHsp* subclasses *HvHsp16.88-CI*, *HvHsp17.5-CI* and *HvHsp17.7-CI*. The *in vivo* relevance of binding specificity of HvHsfB2C transcription factor to HSE-element present in the promoter of *HvSHP17.5-CI* under heat stress exposure is confirmed by gel shift and LUC-reporter assays. Further, we isolated 477 bp cDNA from barley encoding a 17.5 sHsp polypeptide, which was predominantly upregulated under drought stress treatments and also preferentially expressed in developing seeds. Recombinant *Hv*sHsp17.5-CI protein was expressed in *E. coli* and purified to homogeneity, which displayed *in vitro* chaperone activity. The predicted structural model of *HvsHsp-17.5-CI* protein suggests that the α-crystallin domain is evolutionarily highly conserved.

## Introduction

When plants are challenged by drought and temperature stresses, a wide array of interconnected cellular stress response systems is triggered. These cellular responses helps in readjustment of the growth of plants and its survival under abiotic stress exposure. An understanding of the molecular basis of these responses to stress adaptation is essential to make use of them in breeding programs. Heat shock responses (HSR) are temperature-related defense activities and include the induction of evolutionarily conserved chaperone proteins known as heat shock proteins (Hsps). Based on their molecular size, they are classified into different classes i.e Hsp100, Hsp90, Hsp70/DnaK, Hsp60/GroE and small heat shock proteins (sHsps) [1]. Generally, sHsps form large oligomeric complexes [2], ranging in size from 200–800 kDa, and are targeted to different cellular compartments. Though sHsps have been studied in different plant systems [3]; it is not completely known how different sHsps interact with their target proteins and why so many paralogues evolved. Synthesis of sHsps is not specific to heat stress response, but also expressed as part of the developmental program. sHsps are highly expressed in developmental stages like zygotic embryonic tissues, pollen maturation, embryogenesis and during seed maturation [4]–[7]. How these large gene family members of sHsps are finely regulated by a defined set of potential heat shock transcription factors (*Hsfs*) to control many vital processes important during plant development and stress response is largely obscure.

Plant Hsfs have a major role to play in the modulation of transcription during long-term heat shock response [8]. A typical Hsf protein contains a modular structure with an N-terminal DNA-binding domain (DBD), a nuclear localization signal (NLS), a nuclear export signal (NES), and in many cases a less conserved C-terminal activation domain rich in aromatic, hydrophobic and acidic amino acids (AHA) that have been reported to be crucial for activation function [8], [9]. Based on sequence homology and domain architecture, plant Hsfs have been divided into three conserved classes. Several heat shock factor (*Hsf*) complexes could be responsible for the developmental and stress inducible transcription of *Hsp* genes [10]. Rojas et al. [11] demonstrated transcriptional activation of a heat shock protein promoter by ABI3 and Hsf complex. These analyses indicated that genes are controlled by complex regulatory networks [12], [13]. The expression of *Hsps* during different stages of plant ontogeny and its stress-induction depend on the *cis*-motifs present in the respective genes; bound by different transcription factors especially *Hsfs* as demonstrated by transient reporter assays in *Arabidopsis* and sunflower embryos [14], [15]. The expression of particular isoforms of *Hsp* genes during seed development suggests that they may have distinct tissue and cell-specific functions during seed maturation and are regulated by a set of defined developmental programs. Though the importance of *Hsfs* as regulators of the heat shock response is known, the *Hsfs*-*sHsp* interconnections in plant development with special reference to developing seeds and stress responses remain unknown in cereals.

The present study was taken up to find out the expression profiles of *sHsps* and *Hsfs* in barley since that can ultimately help to identify key regulators promoting developmental events under stress. Here, we report a genome-wide survey of all non-redundant sets of *HvsHsp* and *Hsf* genes in the complex genome of barley, a model crop of tribe Triticeae. This survey provided first holistic insights into the interconnected responses of 20 sHsps and 22 Hsfs gene family members in drought stress response in vegetative tissues and also emphasized its role in seed development of barley. *In silico* motif analysis in the 5′ upstream regions of *sHsp* and *Hsf* genes revealed the presence of a distinct set of transcription factor binding sites (*cis*-elements) interlinking the role of ABA in mediating *Hsf* genes. This is the first comprehensive transcriptomic study that identified the differentially expressed *sHsp* and *Hsf* genes and the coexpressed gene networks involved in seed development and drought stress adaptation in barley. Our gene regulatory network analysis identified *HvHsfB2c* as central regulatory hub of sHsps. Our *in vivo* binding assays confirm that HvHsfB2c binds to HSE cis element in the *HvHsp17.5CI* promoter, its transcript is preferentially regulated under desiccation response. Further, we purified the recombinant *Hv*sHsp17.5-CI protein (expressed preferentially in developing seeds and responsive to stress) to homogeneity and validated its chaperone activity.

## Materials and Methods

### Identification and Annotation of sHsp and Hsf Family Genes

The HarvEST Barley database (http://harvest.ucr.edu/) with 50,000 unigenes was searched for genes that encode proteins of *sHsp* and *Hsf* genes, sequence similarity searches were performed using Blastn and Blastx based on known rice and *Arabidopsis* sequence annotations retrieved from TIGR database (http://rice.plantbiology.msu.edu/) and TAIR database (http://www.arabidopsis.org/Blast/). The corresponding cDNA sequences were extracted from the database and subjected to a comparison with recently available 24K full length barley cDNA database to identify full-length clones [16]. cDNA sequences were translated and searched for conserved domains known from the corresponding rice proteins using NCBI database. Multiple sequence alignment and phylogenetic trees comprising barley and rice sHsp and Hsf full length protein sequences were generated by using ClustalW (DNAstar) program. Accession numbers of all identified genes are indexed in tables 1 and 2. Information about the number of amino acids (AA), molecular weights (M.Wt) and theoretical isoelectric point (pI) of all barley sHsps and Hsfs were predicted by using DNAstar software. Organellar targeting of these proteins were predicted by using pSORT (http://psort.nibb.ac.jp/) and TargetP (http://www.cbs.dtu.dk/services/TargetP/) programs. Information regarding ORF length and intron numbers was confirmed by comparing the respective cDNA and genomic clones. Conserved domains of the sHsp and Hsf proteins in barley were determined by Pfam program and from the existing literature.

**Table 1:** List of Hsf genes involved in different abiotic stress conditions and development of barley.

Name	HarvESTID	Fl_cDNA ID	Affymetrix ID	Full/partial	ORF (bp)	Protein (AA)	M.wt (kDa)	pI	Intron	Chromosome Localization	Morex/Bac contig_ID	5′ upstream region (bp)	Predicted localization
HvHsfA1a	35_17839	AK354917	Contig8225_at	Full	1533	510	56.58			5	HVVMRX83KhA 0005M16_v61_c1	2007	Nucleus
HvHsfA2a	35_7894	AK377082	Contig18295_at	Full	1116	371	41.08	5.61	1(77)	4	Contig_2161641	854	Nucleus
HvHsfA2b	35_9896	AK359122		Full	1257	418	46.06	4.82	1(2446)	2	Contig_40049		Nucleus
HvHsfA2c	35_28323	AK363263		Full	1119	372	42.11	4.9	1(891)	1	HVVMRX83KhA 0052F09_v62_c1	2094	Nucleus
HvHsfA2d	35_48033	AK364288	rbaal35o24_at	Partial						4	Contig_5993		Nucleus
HvHsfA2e	35_23711	AK358148		Full	1083	360	40.24	5.65		2	Contig_2162221		Nucleus
HvHsfA2f		AK372701		Full	1053	350	37.98	4.64		4			Nucleus
HvHsfA4b	35_8623	AK371121	Contig23893_at	Full	1299	432	48.54	5.41	1(543)	3	Contig_41104		Nucleus
HvHsfA4d	35_9620	AK362315	Contig18961_at	Full	1296	431	48.72	5.41	1(174)	1	Contig_1009401	2030	Nucleus
HvHsfA3	35_41834	HM446028		Full	1488	495	54.18	6.01	1(1050)	2	Contig_53615	1120	Nucleus
HvHsfA5	35_9627	AK355564	Contig18870_at	Full	1374	457	50.17	5.51	1(1064)	6	HVVMRXALLh A0687I07_v22_c1	1986	Nucleus
HvHsfA9	35_18881	AK362997	Contig10108_s_at	Partial						7			Nucleus
HHsfC1b	35_36864	AK353849	HB03A08_T3_at	Full	708	235	26.07	7.4	1(97)	4	Contig_1017877	798	Nucleus
HvHsfB1		AK366231		Partial						4			Nucleus
HvHsfB2a	35_38788	AK357873		Full	900	299	32.48	5.15		2			Nucleus
HvHsfB2b	35_6843	AK373036	Contig18148_at	Full	1173	390	41.88	5.53		7	Contig_49614		Nucleus
HvHsfB2c	35_18576	AK354133	HV_CEa0014 A18r2_at	Full	1209	402	41.74	4.78		7			Nucleus
HvHsfB4b		AK376881		Full	951	316	34.69	7.11		2			Nucleus
HvHsfB4c	35_31994	AK356002		Full	1167	388	41.42	7.96		2	Contig_221980		Nucleus
HvHsfC1a	35_26831	CB860849		Partial					1(202)	4	Contig_61089	2030	Nucleus
HvHsfC2a		AK369002		Full	801	266	29.48	5.86		4	HVVMRXALLhA 0053M06_v10_c1	2055	Nucleus
HvHsfC2b	35_16479	AK354435	Contig6968_at	Full	801	266	28.27	6.35		7	Contig_57251		Nucleus

The table shows the following details: Harvest unigene ID, full-length cDNA ID, Affymetrix ID, full-length/partial, open reading frame (ORF) size, predicted molecular mass for the deduced proteins, isoelectricpoint (pI), intron number with size, genomic sequence information (Assembly1 Morex ID), derived 5′ upstream of the translational start site and predicted subcellular localization.

**Table 2:** List of sHsp genes involved in different abiotic stress conditions and development of barley.

Name	HarvEST ID	Fl cDNA ID	Affymetrix ID	Full/partial	ORF (bp)	Protein (AA)	M.wt (kDa)	pI	Intron	Chromosome localization	5′ upstream region (bp)	Localization
HvHsp16.9-CI	35_14860	AK362925	Contig2004_s_at	Full	456	151	16.95	5.92		3		Cytoplasm
HvHsp16.88-CI	35_14863	AK355146	Contig2008_s_at	Full	453	150	16.88	5.92		3	1367	Cytoplasm
HvHsp16.86-CI		AK376179		Full	456	151	16.86	5.79		3	2030	Cytoplasm
HvHsp17.5-CI	35_14859	AK250749	HB18H23r_s_at	Full	477	158	17.57	5.9		4	1785	Cytoplasm
HvHsp16.7-CI	35_14864	AK252765	Contig2010_at	Full	453	150	16.76	5.46		4		Cytoplasm
HvHsp17.7-CI	35_1486635_30931	AK368988	Contig2012_s_at	Full	486	161	17.79	5.52		4	2068	Cytoplasm
HvHsp17.3-CII	35_15451	AK375970	Contig3284_x_at	Full	480	159	17.33	6.05		3		Cytoplasm
HvHsp17.7-CII	35_15454	BF263847	Contig3287_x_at	Full	489	162	17.7	6.07		3	2446	Cytoplasm
HvHsp-17.77CII	35_15456	AK355146	Contig3289_at	Full	489	162	17.77	6.41		3	1791	Cytoplasm
HvHsp19.0-CIII	35_18565	AK360636	Contig10029_at	Full	537	178	19.05	6.92		3		Cytoplasm
HvHsp17.1-CV	35_5795,35_27788	AK373216	Contig13073_at	Full	468	155	17.14	6.69	1(92)	2	2078	Cytoplasm
HvHsp17.76-CIX	35_5001	AK370196	Contig11961_at	Full	498	165	17.65	4.68		3		Cytoplasm
HvHsp19.2-CX	35_5077	AK370937	Contig15445_at	Full	534	177	19.26	7.53		6	2285	Cytoplasm
HvHsp15.1-Px		AK365443		Full	417	138	15.14	8.48		4	2000	peroxisome
HvHsp21.9-ER		AK374148,AK374905		Full	618	205	21.97	6.64		6	2100	endoplasmic reticulum
HvHsp-ER	35_31108	BQ766746	EBro08_SQ007_C01_at	Partial						3		
HvHsp21.3-MI	35_16753,35_16752	AK370932	Contig6559_at	Full	582	193	21.32	6.6		7		mitochondria
HvHsp26.8-P	35_21394	AK376976	EBem05_SQ003_L06_at	Full	735	244	26.8	6.1	1(102)	4	763	chloroplast
HvHsp21.2	35_23844	BF623486	Contig21040_at	Full	585	194	21.25	9.31		4		
HvHsp	35_42702	CA005065	HU11O14u_at	Partial						1		

The table shows the following details: Harvest unigene ID, full-length cDNA ID, Affymetrix ID, full-length/partial, open reading frame (ORF) size, predicted molecular mass for the deduced proteins, isoelectricpoint (pI), intron number with size, genomic sequence information (Assembly1 Morex ID), derived 5′ upstream of the translational start site and predicted subcellular localization.

### Expression Analysis Using the Barley Genechip and Gene Network Analysis

RNA isolation of flag leaf and developing seed tissue from control and drought stress treatments was performed as described previously [17]. Probe synthesis, labelling and hybridization were performed according to manufacturer's instructions (Affymetrix). The purified labelled cRNA samples prepared from various vegetative tissues, as well as flag leaf and developing grains collected under control and terminal drought were hybridized to Barley1 GeneChips as described by Close et al. [18]. Arrays were scanned on a GeneChip Scanner 3000. The raw gene expression data of flag leaf and developing grain under control and terminal drought collected from this study were normalized together with publicly available Affymetrix gene expression data obtained from drought-challenged seedlings (series GSE3170), drought stressed 21-day-old plants (series GSE6990) and awn, lemma and palea tissues collected during terminal drought (series GSE17669). The fold change calculations (control versus drought stress) derived from log transformed normalized expression data from every individual stage of plant development were extracted for sHsp and Hsf gene family members and shown in a heat map. Further, to create gene expression atlas from plant ontogeny all the publicly available gene expression covering various tissues and developmental stages from seed germination, seedling establishment, plant maturity, reproductive tissues and developing endosperm and embryo tissue during seed development were normalized using the CEL files in an R package. Log transformed quantile normalized expression values of *sHsp* and *Hsf* gene family were shown in a heat map. Using high throughput coexpression data covering the entire plant ontogeny of barley, the gene regulatory networks have been derived. Gene coexpression network of *HsfB2c* and *HvsHsp17.5* is derived from Plant Network database using Heuristic Cluster Chiseling Algorithm [19]. The gene network vicinity of *HsfB2c* and *HvsHsp17.5* are enriched with various sHsp genes as well other primary metabolism functional categories of coexpressed genes. Further, we used cornet database to predict the protein-protein interactions and coexpression network of AT3G46230 (orthologue of *HvsHsp17.5*) [20].

### *In Silico* Promoter Analysis

To analyze putative *cis*-elements in the promoter region of *sHsp* and *Hsf* family genes, 1,500 bp DNA sequence up-stream of the 5′ end of the cDNAs was extracted from Whole Genome Shotgun sequencing of barley [21] using the viroblast database (http://webblast.ipk-gatersleben.de/barley/index.php). The sequences were further analysed by different web based softwares like PLACE [22] and PlantCARE [23] as well as motifs extracted from the literature. To find out the regulatory *cis* elements, whole promoter sequence was searched in both forward and reverse strands.

### Electrophoretic Mobility Shift Assay (EMSA)

The coding sequence of *HvHsfB2c* was amplified from barley cv. Golden Promise leaf cDNA and transferred to pTOPO-cloning vector (Primer used for amplification: Forward TACCATGGGCAGCAGCCATCATCATCATCATCACAGCAGCGGCCTGGTGCCGCGCGGCAGCCA, Reverse: TACCATGGGCCTCACCTCGAGTTGGACCTGTCCTG). After sequencing of the transferred amplicon translation, HsfB2c protein was synthesized using the PURExpress *in vitro* system from NEB according to manufacturer's protocol. EMSA has been performed as described previously [24] in the presence of 100 ng pdIdC/rn.

The following Oligonucleotides, containing the HSE-binding box were used for the binding reactions: HSE-1: TCGAACAACCCAAAAT CCAAAAAATTCCACAACCCCAAAAAGGC, HSE-2: TCGAGCCTTTTTGGG GTTGTGGAATTTTTTGGATTTTGGGTTGT.

### Transient expression of HvHsfB2c-derivatives

Arabidopsis Col-0 mesophyll protoplast isolation and transformation was carried out with plants grown in soil under controlled conditions in a phytochamber for 4 weeks (8 h light/16 h dark at 20°C and 18°C, respectively) [25]. For microscopic localization studies, HvHsfB2c was cloned into pENSG-/pEXSG vectors (N-/C-terminal fusion with CFP, respectively) and 10 µg of DNA per 100 µl protoplasts was transformed. After 16 h incubation in the dark, CFP fluorescence was evaluated using the LSM 710 Laser Scanning System (Zeiss, Oberkochen, Germany). In case of the luciferase reporter assay, a *HvsHsp17.5-CI* promoter-luciferase construct, pUBQ10-GUS for normalization [26] and either pEXSG-HvHsfB2c or pUGW15-CFP [27] as control were transformed (10 µg total DNA per 100 µl protoplasts; ratio 1∶1∶1). Heat stress mimicking condition was applied by temporal increase of the incubation temperature for 10 min to 35°C. The used *HvsHsp17.5-CI* promoter fragment contained the region 700 bp upstream of the start ATG. Primer used for amplification are Forward: pHsp17.5_BamHI CAGGATCCTGTTGAGGACTGACA, Reverse: pHsp17.5_NcoI CACCATGGCGATCGGGTACTCGG. The luciferase-assay was performed as described in [28].

### Homology Modeling of *Hv*sHsp17.5-CI

*Hv*sHsp17.5-CI molecular model was generated using the homology modeling server SWISS-MODEL [29] utilizing *Triticum aestivum* sHsp16.9 protein crystal structure as a template (PDB No:1gmeA). Following PROCHECK analysis, the model with the best Z-score −0.92 showed an RMSD of 2.70 Å and 70% sequence identity with respect to the template. The modeled residue range was taken from amino acids 2–158 by I-TASSER. Dimeric structure was generated by aligning the monomeric structure with the α-crystallin domain of *Ta*sHsp16.9 and *Hv*sHsp17.5-CI using the program I-TASSER server [30], [31] with the best C- score 0.897, an RMSD of 2.40 Å and 0.699 TM-score with respect to the template.

### Expression and Purification of *Hv*sHsp17.5-CI Recombinant Protein

*Hv*sHsp17.5-CI specific oligonucleotide primers were designed one for the N-terminus region (5′-ATACTACATATGTCGCTGATCCGTCGCAGCAACGT-3′) and the other for C-terminus region (5′-TAATGCGGCCGCCTAGCCGGAGATCTGGATGGAC-3′). The 5′ and 3′ untranslated regions in the cDNA were removed and an *Nde*I site at the translation initiation and a *Not*I site just downstream of the translation termination codon were introduced. *Hv*sHsp17.5-CI PCR amplified cDNA product was digested and cloned into *Nde*I and *Not*I sites of pET28a (+) expression vector. The sequences adjoining the 5′ and 3′ ends of the cloned segment were confirmed by sequencing. This construct resulted in the expression of *Hv*sHsp17.5-CI polypeptide with additional extra 20 amino acids including hexa histidine tag at the N-terminus. The recombinant pET28a-*Hv*sHsp17.5-CI plasmid was transformed into BL21 (DE3) cells and grown in LB-medium supplemented with 50 µg/ml kanamycin at 37°C. As absorbance at 600 nm (A_600_) reached a value of about 0.5–0.6, the expression of recombinant *Hv*sHsp17.5-CI was induced by adding IPTG (isopropyl β-D-1-thiogalactopyranoside, 1 mM) and the cells were allowed to grow for an additional period of 3 h at 37°C. After induction of recombinant protein, *E. coli* cells were lysed by sonication. Native recombinant *Hv*sHsp17.5-CI protein was purified from clarified *E. coli* lysate through Ni-NTA column chromatography, following the manufacturer's instructions (Qiagen, Germany) and protein samples were analyzed by SDS-PAGE.

### Stress Tolerance of *E. coli* Overexpressing Recombinant *Hv*sHsp17.5-CI and its Chaperone Activity

*E. coli* BL21 (DE3) cells transformed with pET28a (+) (vector control) or with pET28a-*Hv*sHsp17.5-CI plasmids. Transformed *E. Coli* were grown overnight in fresh LB medium containing 50 µg/ml kanamycin. When the absorbance at 600 nm reached a value of 0.25, varying concentrations of NaCl (0–750 mM) for salinity stress, and 0–25% of polyethylene glycol (PEG, molecular weight of 3,350) were added to impose dehydration stress after the addition of IPTG. For temperature stress, cultures were grown at 37 to 55°C after IPTG treatment. After induction with the addition of IPTG (1 mM), cultures were kept at 37°C for 12 h in a shaking incubator. Cell growth was monitored by measuring the absorbance at 600 nm. Each experiment was repeated thrice and average readings were taken. Recombinant *Hv*sHsp17.5-CI chaperone activity was assayed by using thermo labile restriction enzyme, *Swa*I (New England Biolabs, Beverly, MA) as previously described [32], [33]. The heat-labile *Swa*I enzyme was pre-incubated at a range of temperatures (25, 30, 35, 40, 45 and 50°C) for 60 min in the presence of either BSA (5 µg) or recombinant *Hv*sHsp17.5-CI (5 µg). After pre-incubation, the reaction mixture was cooled to 25°C and plasmid DNA (500 ng) with a unique *Swa*I recognition site was added and further incubated at 25°C for 60 min for restricting digestion of plasmid DNA. The restriction digested plasmid DNA samples were separated by electrophoresis on 1% agarose gel and stained with ethidium bromide. Plasmid digestion profiles were compared with their respective controls.

## Results

### Identification of *sHsp* and *Hsf* Gene Families in Barley

The conserved amino acid sequence of α-crystallin domain (ACD) for sHsps and DNA binding domain (DBD) for Hsfs was adapted as a query to search possible homologs encoded in the barley genome using HarvEST (http://harvest.ucr.edu/) and NCBI databases. This resulted in the retrieval of 20 *sHsp* and 22 *Hsf* encoding gene sequences. Further, functional annotations of these sequences were verified by using BlastX, BlastN and BlastP programmes of NCBI. Details of all the genes encoding for barley *sHsps* and *Hsfs* are represented in the Tables 1 and 2 respectively. Comparative sequence alignment of the genomic and cDNA sequences of *sHsp* and *Hsf* genes revealed the predicted exons and introns. Only certain classes of sHsps possess introns. *Hv*sHsp proteins showed variation in length (from 138 to 244 amino acids), isoelectric point (pI) values (4.68 to 9.31) and molecular weights (15.14 to 21.25 kDa). Prediction of subcellular localization of these proteins using pSORT and TargetP programmes [34]–[36] indicated that 13 sHsp proteins are located in the cytoplasm and one each in the mitochondria, ER, plastid and peroxisome. In case of Hsf family proteins, 21 are distributed in the nucleus and one in the cytoplasm.

### Phylogenetic Analysis of *Hsf* Gene Family Members

**Figure 1:**
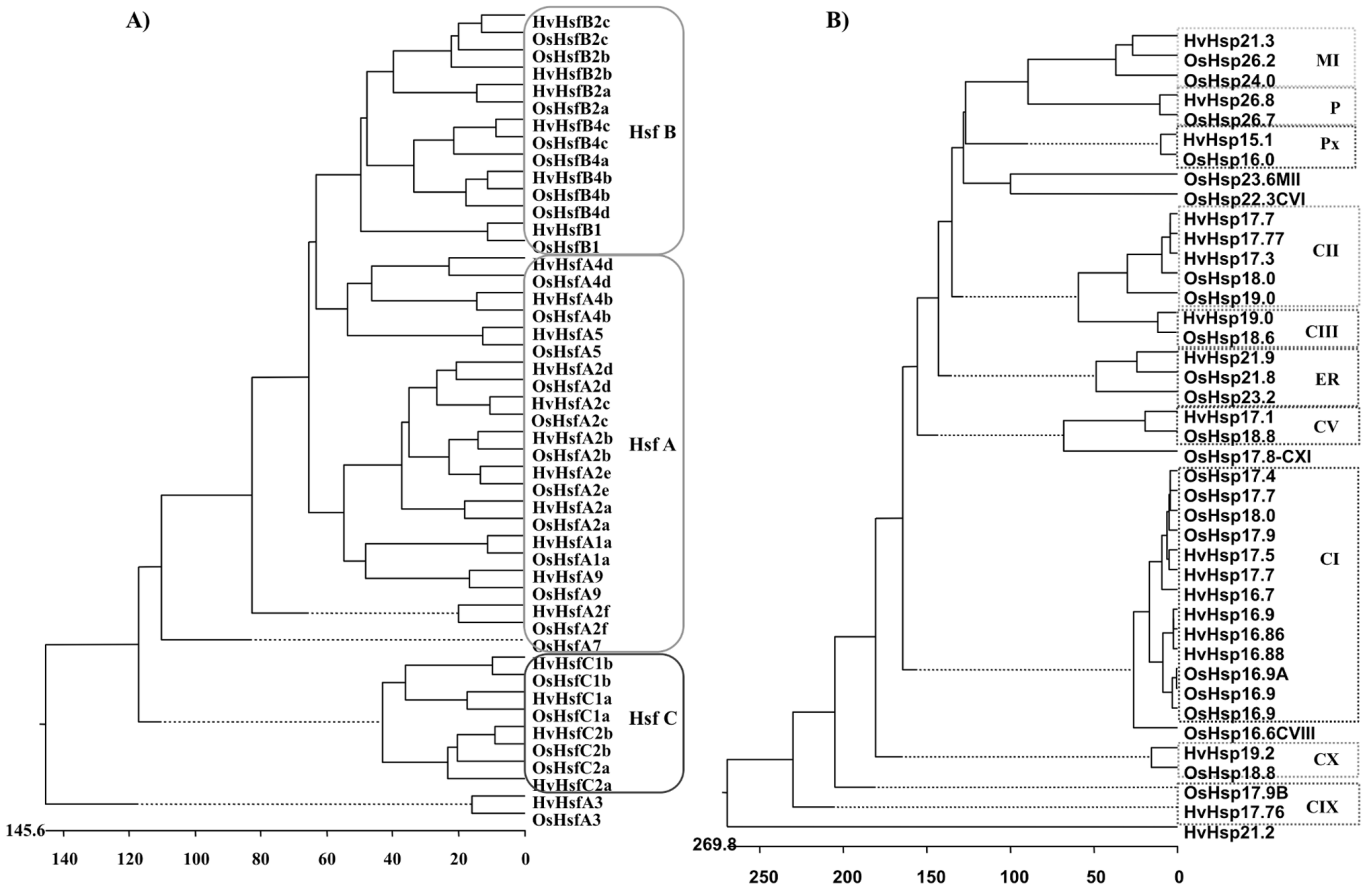
Phylogenetic relationships between barley and rice sHsp and Hsf proteins. The phylogenetic tree was drawn from the deduced amino acid sequences of sHsp (A) and Hsf (B) proteins from the barley and rice genome using the ClustalW (MegAlign, DNAStar). Subfamilies are shaded in different colours.

A phylogenetic tree was constructed for 22 barley and 25 rice *Hsf* genes. All *Hsfs* clustered broadly into three major cluster groups A, B and C, which included representative genes of barley and rice (Figure 1A). The major cluster class A is further divided into several subclasses based on its phylogenetic relationship and designated as A1, A2, A3, A4, A5 and A9. *HvHsfA3* and its rice ortholog *OsHsfA3* did not cluster with class A but grouped separately (Figure 1A). This however appears to be closer to *HsfC*. All class B *Hsfs* showed divergence from a common point but are closer to *HsfA* class genes compared to class C (Figure 1A). The motif distribution also followed the same scenario with the phylogenetic analysis. Therefore, it looks from the phylogenetic tree that one subgroup of class A *Hsf* gave rise to class B and C (Figure 1A).

The detailed knowledge of tomato, rice, maize and *Arabidopsis* Hsf functional domains and motifs enabled us to analyze similar kind of domains for the 22 Hsfs identified in the barley genome (Table 1 and Table S1). Motif analysis and sequence alignment of *Hv*Hsfs showed a highly structurally conserved DBD domain which contained 3 α-helix bundles and 4 β-strands in the N-terminal region (Figure S1) as described earlier in other plant species [37]–[39]. Hsfs function as transcriptional activators because of AHA motif characterized by aromatic (W, F, Y), large hydrophobic (L, I, V) and acidic (E, D) amino acid residues in their C-terminus (Figure S1A). However, class B and C putative AHA motifs could not be predicted in barley. Due to the absence of AHA motif in class B and class C subfamilies, they probably lack activator function. The C-terminal activation domain was found only in *HsfA*, suggesting that only class A *Hsfs* can activate autonomously.

### Phylogenetic Analysis of *HvsHsp* Gene Family Members

We performed phylogenetic analysis using the deduced amino acid sequences of different isoforms of barley and rice sHsps. The sHsp sequences were clustered into different groups based on their subcellular localization (Figure 1B). Most of the genes from barley and rice fell into the same sub-clusters, which indicated that they are highly conserved. Using rice sHsp family classification, we identified sHsp subfamilies in barley and annotated 20 sequences. These are distributed into 10 different classes, including cytoplasmic (CI, CII, CIII, CV, CIX and CX), mitochondrial (MI), ER, plastidial (P) and peroxisomal (Px) proteins. While 6 proteins have fallen into CI, 3 into CII and one each into the remaining classes (Figure 1B). Furthermore, one sHsp gene named *HvsHsp21.2* stood apart and was not clustered with any of the sHsp genes from rice. Its subcellular location is also not known. The CI gene family is generally the largest sHsp subfamily in rice and *A. thaliana*, it has 7 and 6 gene members respectively. The phylogenetic relationships within the cytosolic I subfamily deserves particular attention. In barley, there are two sub-clusters of cytosolic I genes (Figure 1B). One cluster comprising of *HvsHsp16.9*, *HvsHsp16.86* and *HvsHsp16.88* members, closely related to *O. sativa 16.9A*, *16.9B* and *16.9C* and the other cluster members *HvsHsp17.7*, *HvsHsp17.5* and *HvsHsp16.7* clustered to *O. sativa 17.4*, *17.7*, *17.9A* and *18.0*. While all the *Os16.9 sHsps* are located on chromosome 1, *Os17.4* is located on chromosome 3. The six sHsps of barley have been analyzed for their chromosomal localization. While sHsps 17.5, 17.7, 16.7 are localized on chromosome 4 in barley, other sHsps 16.9, 16.86 and 16.88 are located on chromosome number 3 (Table 2). The number of sHsp gene family members in the cytosol is larger compared to other cell organelles, indicating that cytosol might be the primary site of action for the function of sHsps. The pattern distribution among different classes also suggested that this small Hsp gene family is highly conserved among cereals. Sequence homology among these different classes ranged from 62 to 80%, but the functional relationships of these individual subfamilies to each other is not clear.

Sequence alignment of *sHsp* subfamilies revealed some interesting patterns of sequence conservation. The main characteristic feature of all the sHsps is the presence of an evolutionarily conserved central domain of 80–90 amino acids named α- crystallin domain (ACD) but have divergent N- and C-terminal extensions. N-terminal preceding the ACD region displayed variability in length and amino acid composition that contributed to a large extent for the structural diversity among subfamilies of sHsps [40]. The ACD region further can be divided into consensus I and II domains separated by a hydrophilic domain of variable length. All *HvsHsps* shared a consensus region I, which is highly conserved throughout the eukaryotes but the second consensus region II is unique and conserved only in plants [40] (Figure S2). The residues Pro-X (14)-Gly-Val-Leu in consensus region I are a conserved signature motif present in almost all sHsps. A similar motif Pro-X (14)-X-Val/Leu/Ile-Val/Leu/Ile also appeared in the consensus region II [41]. Outside the α-crystallin domain, a typical “I/V-X-I/V” motif in the C-terminal extension can be recognized in most sHsps except in class V (Figure S2D). Arginine residue present in ß7 strand among sHsps represented the most conserved site in the eukaryotes. This arginine in barley is located at the same position as in the case of α-crystallin structure of wheat (Figure S2). The class I cytosolic proteins have a consensus region of 15 amino acids at the N-terminus (Figure S2A), class II have 11 amino acids (Figure S2B) and class P proteins comprises 24 amino acids which do not exist in other classes (Figure S2J). The amino acid similarity between individual sHsps belonging to different groups range from 62% to 80%, whereas the similarity between individual sHsps within the groups ranged from 85% to 99%. However, there are a number of secondary structural features that are conserved across sub families irrespective of the species.

### Differential Expression of *sHsp* and *Hsf* Genes During Plant Development and Drought Stress Response

**Figure 2:**
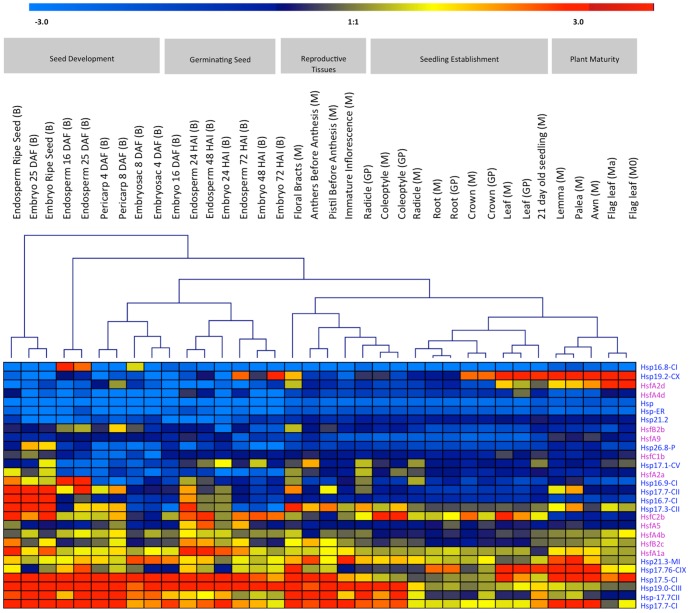
Expression profiles of sHsp and Hsf family genes during various stages of plant ontogeny analyzed by the Affymetrix 22K barley gene chip. Horizontal rows represent expression patterns of individual gene. Trivial names of genes as well as the corresponding Affymetrix IDs are given. Vertical lines represent the developmental stages and investigated tissues. Signal intensities: red, high expression; yellow, moderate expression; blue, low expression. Represented cultivars are named as M, ‘Morex’; Mo, ‘Morocco’, Ma ‘Martin’, B, ‘Barke’; GP, ‘Golden Promise’. Quantile normalized expression values are given as log2.

Unraveling the co-expression patterns can render important clues regarding the gene function. To understand the potential interlinking role of *sHsp* and *Hsf* genes, we monitored the expression profile (a) during plant ontogeny and (b) in response to drought stress. Microarray data revealed that *sHsp17.7* (*CI and CII*), *17.5* (*CI*), and *19* (*CIII*) are mostly expressed in developing seed tissues like endosperm (25 DAF and ripe seed), embryo (25 DAF), pericarp (4 DAF) and in reproductive tissues such as mature floral bracts (Figure 2). The expression of these genes was however, less obvious during seedling establishment in tissues like coleoptile, root and crown. Among *Hsfs*, *HsfA1a*, *HsfA2a*, *HsfB2c*, *HsfC2b* and *HsfA4b* were more preferentially expressed in endosperm and embryo (25 DAF) than others (Figure 2), suggesting a tighter coexpression with *sHsp17.7* (*CI* and *CII*), *17.5* (*CI*), and *19* (*CIII*). These specific gene family members of *sHsp* and *Hsf* genes are regulated by a defined developmental program such as embryogenesis and seed maturation events.

**Figure 3:**
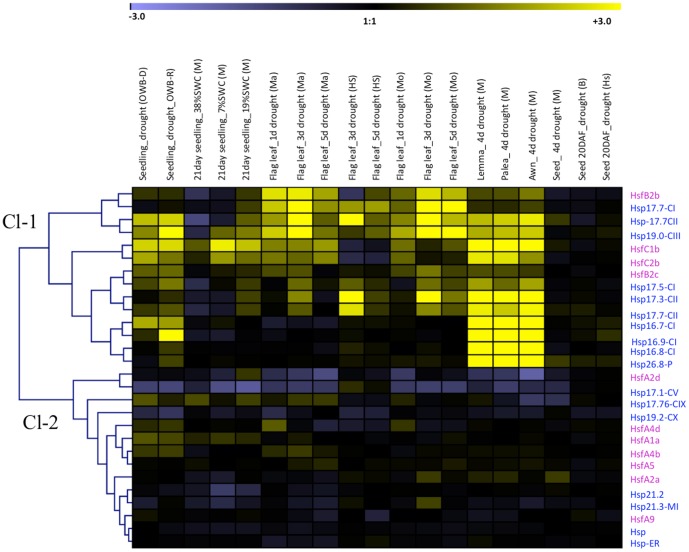
Expression profiles of barley genes responsive to drought. Expression ratios (drought vs control) are calculated based 3 replications. Fold change values are colour-coded: dark yellow >6 fold up-regulated, black no change, violet >6 fold down-regulated. Horizontal rows represent gene expression patterns. Vertical lines represent different stress treatments. Gene expression data refers to cvs. Brenda (B), Morex (M), Morocco (Mo), Martin (Ma), Oregon Wolf Barley-Dominant (OWB-D), Oregon Wolf Barley-Recessive (OWB-R), Hs (*H. spontaneum* HS584).

Interestingly, many sHsps in barley which are highly expressed in developing seed were also found to be preferentially upregulated under drought in vegetative tissues. The clustering process identified the genes that were highly up-regulated under drought stress in different stages of the plant development (cluster-1) and other gene sets were down regulated in majority of the stages with different time points (cluster-2) (Figure 3). Genes included in the cluster-1 are both sHsps (*HvHsp17.5-*CI, *17.7-*CI, *17.7*-CII, *19*-CIII, *17.3*-CII, *17.77*-CII, *16.7*-CI, *16.9*-CI, *16.8*-CI and *26.8-P*) and Hsfs (*HvHsfB2b*, *B2c*, *C1b* and *C2b*). They are up-regulated under drought in all the developmental stages (early seedlings, 21-day-old seedlings, flag leaf, lemma and palea) and also abundantly expressed in a range of organs (floral bracts, pistils before anthesis, 5-day-old caryopsis and 22 DAP embryo) under normal conditions (Figs. 2 and 3). These genes perhaps have a protective chaperone role both during critical stages of plant development as well under drought. Thus, cluster 1 genes could be considered to represent as a core set of drought responsive genes. Within cluster 2, several sHsps (*HvHsp17.1*-CV, *17.76*-CIX, *19.2*-CX, *21.2*, *HvHsp-ER*, *21.3 MI* genes) and *Hsf A1a*, *A2a*, *A2d*, *A4b*, *A4d*, *A5* and *A9* members were either down-regulated or non-differential regulation in most of the developmental stages of plant during water stress or their expression was low.

### *In Silico* Analysis of sHsp and Hsf Family Promoter Regions

The regulatory *cis*-acting transcription factor binding sites in the sHsp and Hsf promoters were identified using PlantCARE and PLACE databases and details are depicted in Figure S3 and Figure S4. The *cis*-motif ABRE [42] required for ABA response is present in the promoters of all the Hsf genes except in *HsfA2c*, suggesting that these *Hsf* genes are involved in ABA mediated signal transduction. The HSE motifs responsible for the expression of *Hsp* genes during high-temperature stress are present in the promoter regions of many *sHsps* (*HvHsp16.86-CI*, *17.5-CI*, *17.7-CI*, *17.77-CI*, *17.1-CV*, *19.2-CX*, *26.8-P*), as well in *HsfA* class members (*HsfA1a*, *A2a* and *A2c* genes). Besides, within barley promoter sequences of *sHsp* and *Hsf*, many motifs which are associated with abiotic stress responses (heat shock element, HSE; drought-inducibility, MBS; low temperature responsive, LTR; anaerobic induction, ARE), hormonal responses (MeJA-responsiveness, TGACG-motif) and seed development (Skn-1 motif and GCN4 motif) are enriched (Figure S3 and Figure S4).

Among all, *Hsp17.5-CI*, *17.77-CII* and *HsfA2a* have large number of different *cis*-motifs, related to seed development and drought stress. Thus, it is expected that many of these *sHsp* genes are found to be regulated both under stress as well as during seed development, perhaps due to common physiological cause of desiccation related events. Notably, many *HvsHsp* and *Hsf* genes containing Myb binding site (MBS) element in the upstream region (Figure S3 and Figure S4), which has a role in desiccation stress response, were found to be inducible by drought stress treatment in a tissue specific manner (Figure 2, 3). Seed development specific motifs such as Skn-1 and GCN4 conferring endosperm specific expression were also found in many *sHsp* and *Hsfs*. Seven Skn-1 motifs were noticed in *HvsHsp17.1-CV*, 4 in *17.7-CII*, 5 in *HsfC2a* and 4 in *A5* (Figure S3 and Figure S4) and displayed the highest expression in endosperm and embryo compared to other plant parts (Figure 2). In addition, *cis*-motifs like CCGTCC, TGA and ARE/GC related to meristem expression, salicylic acid and anaerobic inductions were observed in most of the *HvsHsp* and *Hsf* genes. Such a tissue specific expression of heat shock genes reveals important developmental role in the reproductive tissues during development.

### Gene Network of *HvHsfb2c* Based on Genome-Wide Coexpression Data

**Figure 4:**
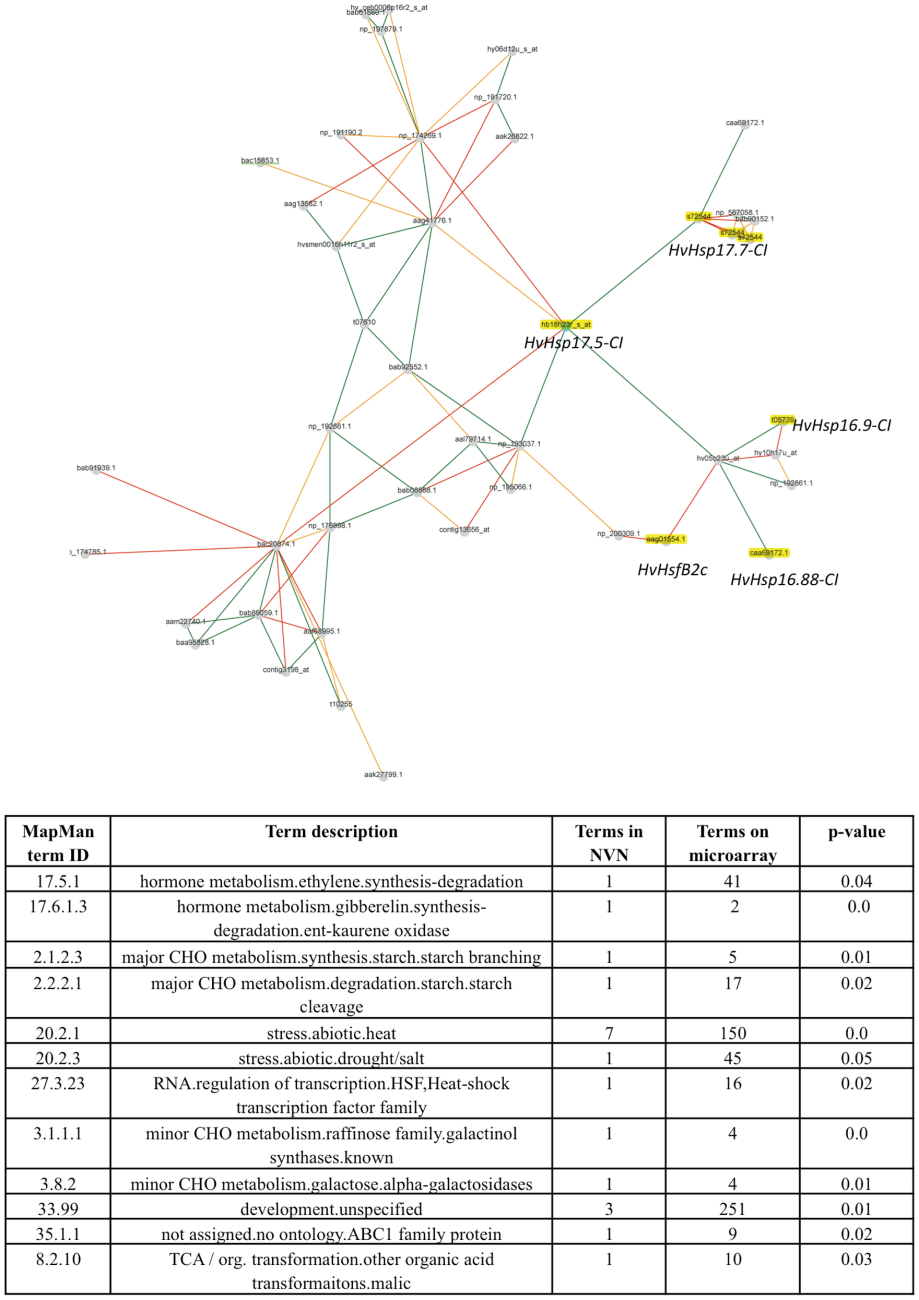
Gene network analysis. Gene co-expression network of *HvHsfB2c/HvHsp17.5-CI*, is derived from Plant Network using Heuristic Cluster Chiseling Algorithm based on genome-wide plant ontology high throughput gene expression data. Meta-network containing genes of *HvHsfB2c* cluster are enriched for several sHsps (highlighted in yellow colour) and also enriched Hsp class in the MapMan functional categories are represented (see table). For further details refer Table S1.

**Figure 5:**
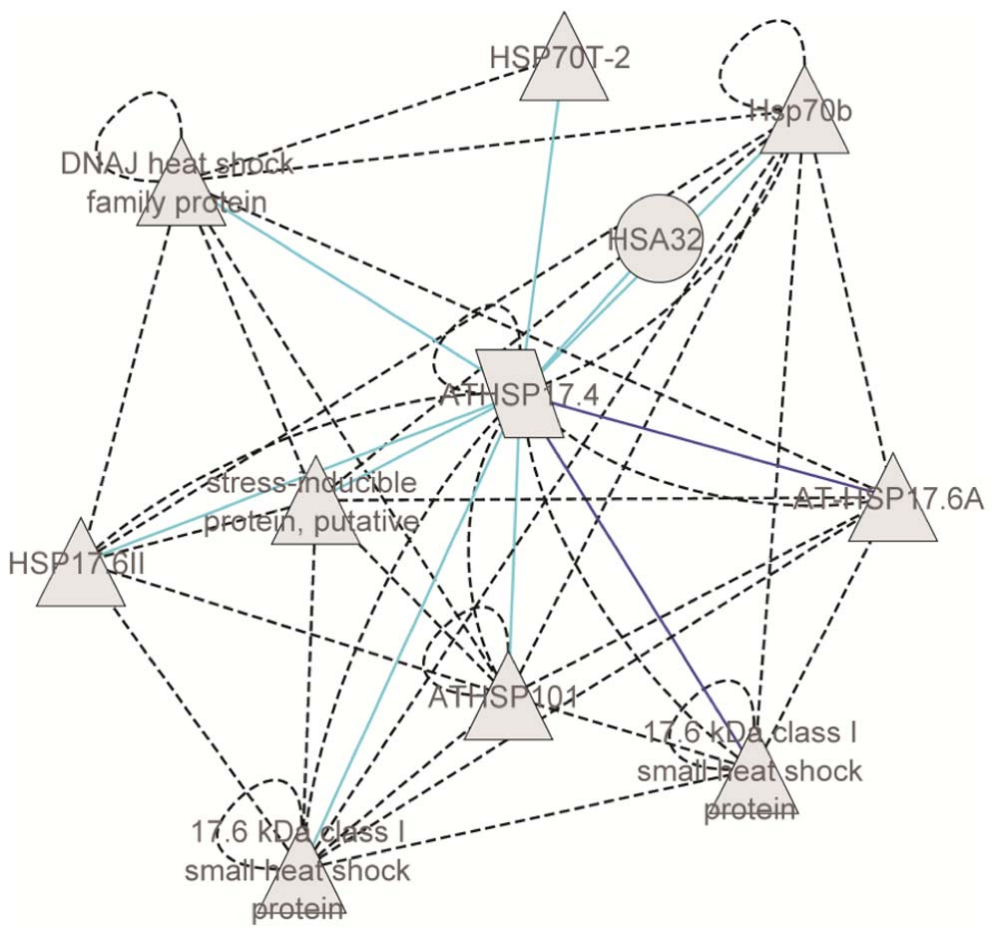
Integrative view of protein-protein interactions and coexpression networks of AT3G46230 (orthologous gene of *sHsp17.5* in barley) derived in *Arabidopsis* based on CORNET correlation networks [**20**]. Predicted protein interactions are highlighted in dotted black colour and also autoregulatory loops are shown for several Hsp proteins. The embedded coexpression network of AT3G46230 includes several direct and indirect targets identified through microarray experiments from 256 experimental data sets generated from abiotic stress treatments. Significance of coexpression is measured by the Pearson correlation coefficient (dark blue lines represent positive correlation of 0.9; light blue lines represent positive correlation of 0.8).

In contrary to *Arabidopsis*, *HvHsfB2c* possess a nuclear signal and was preferentially expressed in developing seeds as well prominently upregulated under drought. Our genome-wide gene expression data covering plant ontogeny suggest that *HvHsfB2c* is the central hub in the derived gene network (Figure 4). Network data also emphasize that *HvHsfB2c* is coexpressed in the central hub of several small Hsp members (*HvHsp16.9-CI*, *HvHsp16.88-CI*, *HvHsp16.87-CI*, *HvHsp17.5-CI*, and *HvHsp17.7-CI*), which are preferentially expressed in developing and imbibed seeds (Figure 4 and Table S2). Also, *HsfB2c* is coexpressed under drought stress together with several *HvHsp* CI, CII and CIII family members. Thus, *HsfB2c* transcription factor is likely to play an important role in both events (a) desiccation tolerance during seed maturation and (b) drought tolerance in possibly regulating the expression of several subclasses of *Hv*Hsp. Further, we performed cornet analysis to unravel the potential protein interaction partners and coexpressed gene regulatory networks of AT3G46230 (orthologous gene of *sHsp17.5* in barley) in *Arabidopsis*. The predicted protein-protein interaction analysis suggests that AT3G46230 is likely to form protein complexes with several small heat shock proteins Hsp17.4, Hsp17.6 class I, Hsp17.6 class II, Hsp 17.6A and Hsp70 family (Figure 5).

### Transcriptional Regulation of HvsHsp17.5-CI by Hv*HsfB2c* under heat stress

**Figure 6:**
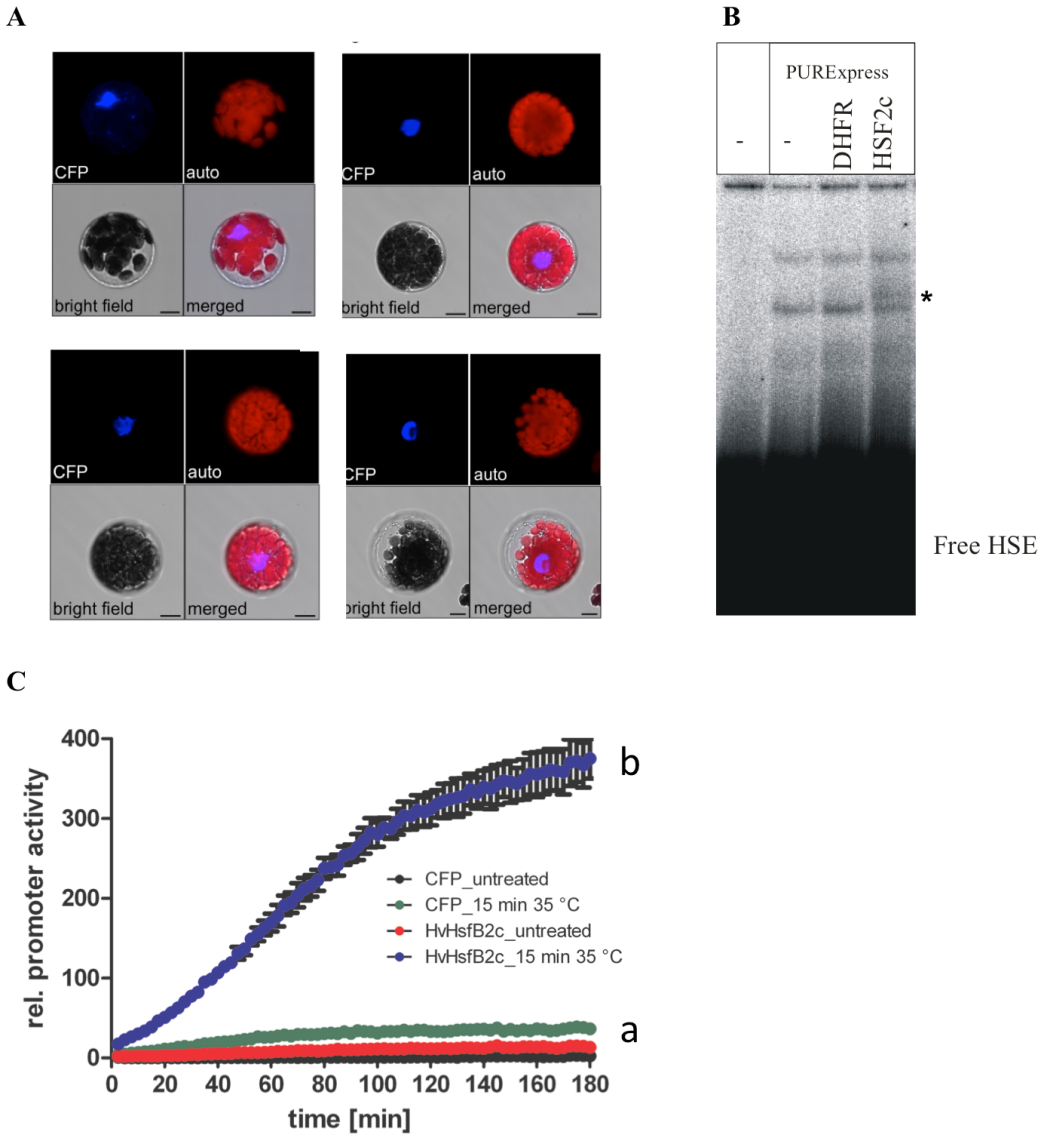
Transcriptional regulation of *HvsHSP17.5-CI* by HvHSF2c under heat stress. A. Subcellular localisation of HvHsfB2c-CFP in transient transformed *Arabidopsis thaliana* mesophyll protoplasts, scale = 10 µM. B. EMSA with *in vitro* translated HvHsfB2c SFB2c on HSE-box from the *ProHvHsp17.5-CI*. Extract of PURExpress without template DNA and translated DHFR (*E.coli* dihydrofolate reductase) were included as negative controls. * indicates HsfB2c specific band. C. Luciferase reporter gene assay. *ProHvHsp17.5::LUC* was used as reporter gene in *Arabidopsis thaliana* Col-0 protoplast co-transformation experiments with HvHsfB2c expression vector at 35°C (blue line, a: significant difference to controls), (b) CFP-control vector at 21°C (black line), HvHsfB2c expression vector at 21°C (red line) and CFP-control vector at 35°C (green line). Results are depicted as LUC/GUS ratios. The experiment was repeated twice in triplicates with similar results. Error bars indicate the standard error of the mean of 3 replicates.

At first, the sub-cellular localisation of N- and C-terminal cyan fluorescing protein (CFP)-tagged *HvHsfB2c* was tested in a transient expression assay using *Arabidopsis thaliana* mesophyll protoplasts. The protein sequence of HvHsfB2c contains two signatures for nuclear localisation (pat4: HRRK at 131 amino acid and bipartite: RRGEKRLLCDIHRRKVV at 120 amino acid). In agreement with WoLFPSORT algorithm [43] the predominant nuclear localisation of HvHsfB2c could be confirmed for the C-terminal (Figure 6A) and the N-terminal CFP-fusion derivatives (Figure S5). As HvHsfB2c was found predominantly in the nucleus, regulation via heat stress induced translocation of the protein is very unlikely. In order to verify the *in silico* predicted binding of HvHsfB2c on *HSE-box* from the *HvsHsp17.5-CI* promoter, Electrophoretic Mobility Shift Assay (EMSA) was performed. For the analysis, a 44 bp DNA fragment was used containing the *HSE-Box* (AAATTCC) as core element. The coding region of *HvHsfB2c* was amplified from cDNA derived from barley *cv.* Golden Promise leaf RNA. As indicated in Figure 6B, the appearance of an additional band in the EMSA study refers to the DNA binding ability of *in vitro* translated HvHsfB2c. For analysis of the binding specificity, luciferase (LUC)-reporter assays were performed (Figure 6C). The LUC-gene was fused to a fragment of the *HvsHsp17.5-CI* promoter containing the region 700 bp upstream of the start ATG including the HSE-element. As indicated by the reporter gene assay no LUC-activity could be detected under control conditions. The presence of the co-transformed HvHsfB2c alone was not sufficient to increase the LUC-reporter gene activity. A strong increase of LUC-activity could be detected in the HvHsfB2c co-transformed mesophyll protoplasts under heat stress conditions (Figure 6C).

### *In Silico* Structural Analysis and Homology Modeling of the *Hv*sHsp17.5-CI Protein

**Figure 7:**
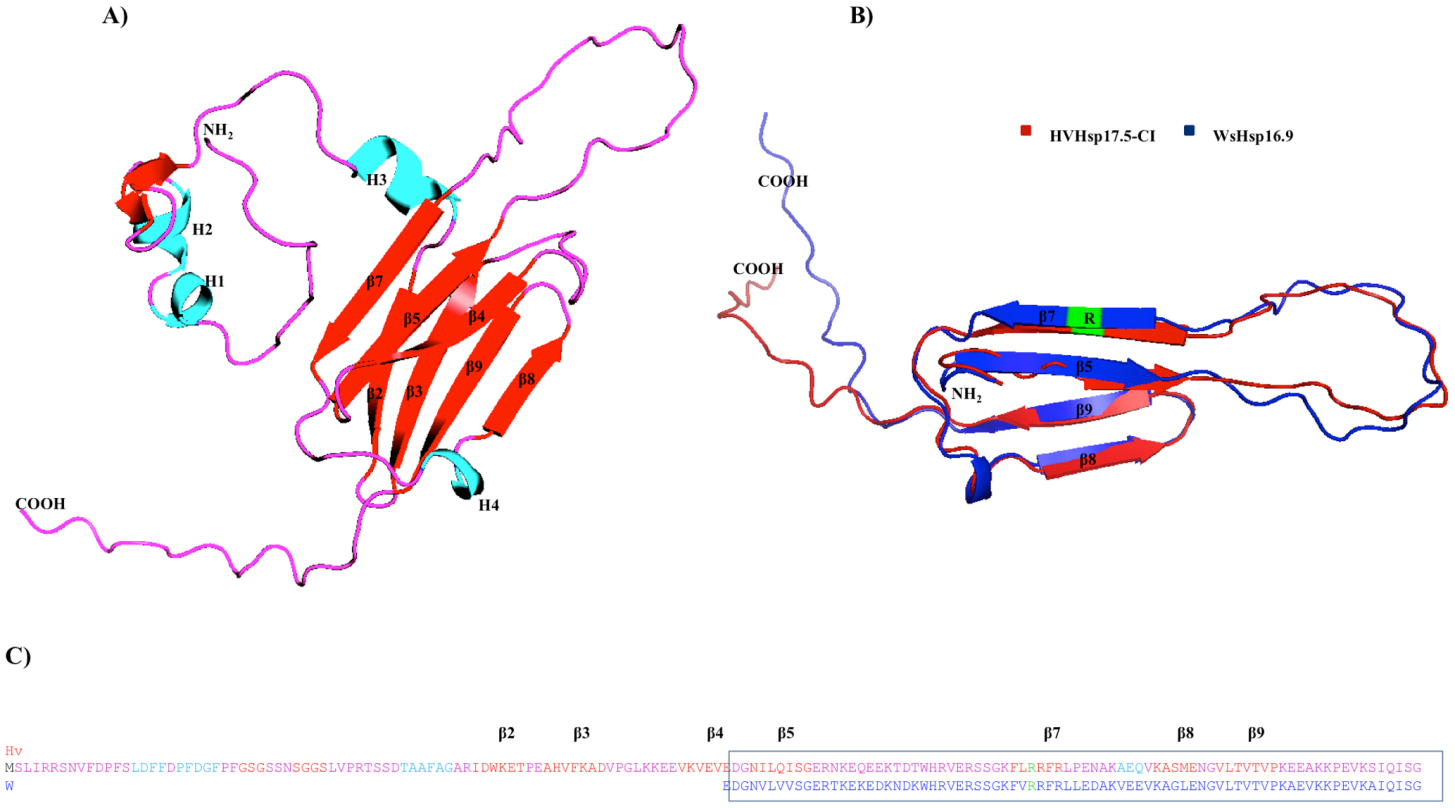
Structural organization of the *Hv*sHsp17.5-CI protein. A. The α-helix and ß-strands held between the two surface loops are shown in red and light blue colors. The N and C termini are indicated by NH_2_, COOH, letters respectively. B. Structural alignment of the crystallin domain of *Ta*sHsp16.9 and *Hv*sHsp17.5-CI proteins are labeled as blue and red respectively. Highly conserved arginine (R) residue is shown in green color. C. Structural alignment of *Hv*sHsp17.5-CI and *Ta*sHsp16.9. The conserved regions of *Hv*sHsp17.5-CI and *Ta*sHsp16.9 are labeled with respective colors of figure A and B.

The crystal structure of *Ta*Hsp16.9 protein (PDB No: 1gmeA) was chosen in the present study as a template for homology modeling of *Hv*sHsp17.5-CI (Figure 7A) using the server SWISS-MODEL [44]. It has been found that the two proteins have an identity of 70% at the amino acid level and a similarity of 81%. Our annotation revealed that the evolutionarily conserved α-crystallin domain contains a compact ß-strand that was responsible for dimer formation while the rest of the protein forms a conserved secondary structure despite large levels of sequence diversity [45]. There are a number of secondary structural features that are highly conserved across subfamilies, such as the ß3, ß4, ß5, ß8 and ß9 (Figure 7A). We also generated dimeric structure alignment by superimposing the monomeric structure with the α-crystallin of barley and crystallin structure of *Ta*Hsp16.9, using the program I-Tasser [30] and showed the dimer interface of both proteins is virtually identical. *Hv*sHsp17.5 and *Ta*Hsp16.9 differed in the length of beta strands but they maintain same number in their conserved “α-crystallin domains” (Figure 7B). Considering the number of β-strands and their positions in the domain of α-crystallin, it is expected that the structure of this domain is closer to that of *Ta*Hsp16.9.

### Prevention of High Temperature-Induced Thermal Inactivation of *Swa* I Restriction Enzyme by Recombinant *Hv*sHsp17.5-CI

**Figure 8:**
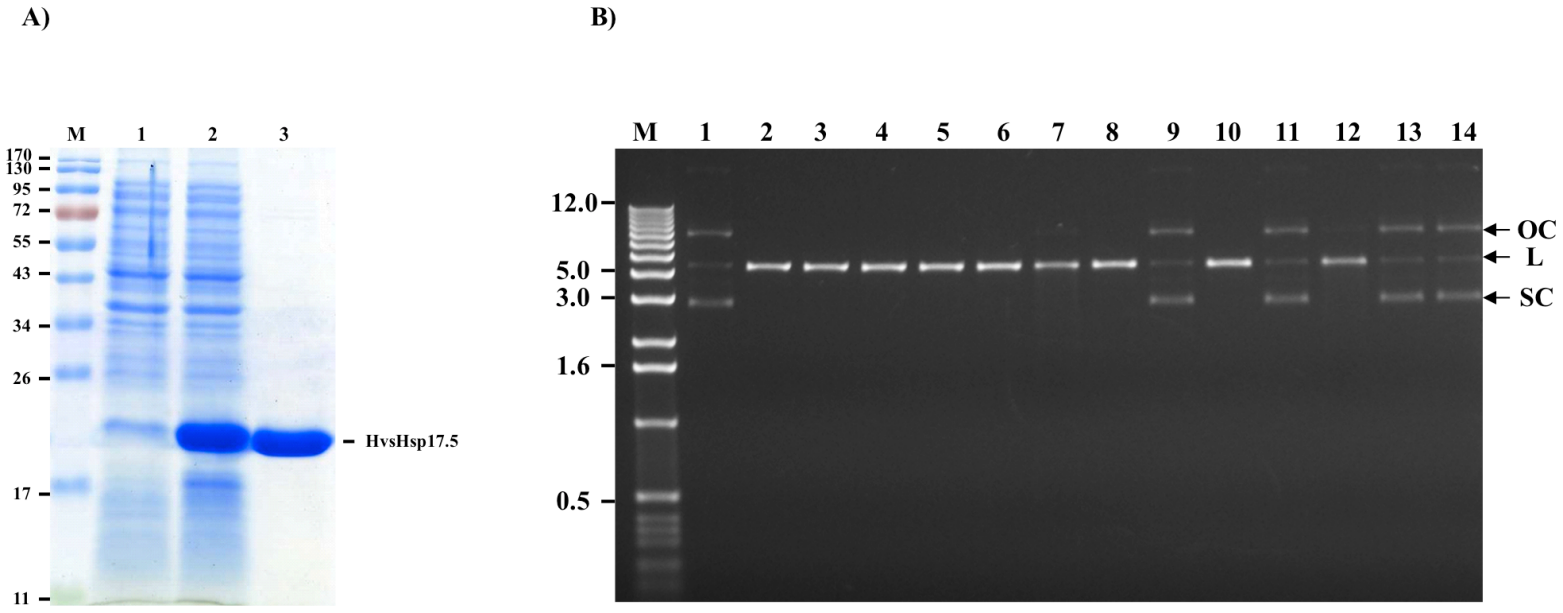
Expression, purification and chaperone activity of recombinant *Hv*sHsp17.5-CI. A) Expression of recombinant *Hv*sHsp17.5-CI in *E. coli*. Lane M, molecular weight marker; lane 1, uninduced; lane 2, induced; lane 3, purified recombinant *Hv*sHsp17.5-CI protein. Figures on the left indicate molecular weight in kDa. B) Prevention of thermal inactivation of *Swa*I restriction enzyme by recombinant *Hv*sHsp17.5-CI. The *Swa*I restriction enzyme was preincubated at 25, 30, 35, 40, 45 or 50°C in the presence of either BSA or recombinant *Hv*sHsp17.5-CI for 60 min. Residual activity of *Swa*I was determined by incubation with 300 ng plasmid at 25°C for 60 min, followed by electrophoresis on a 1% agarose gel. Lane M, 1-Kb DNA ladder; lane 1, plasmid DNA control (without *Swa*I digestion); lane 2, plasmid DNA digested with the *Swa*I restriction enzyme; lanes 3, 5, 7, 9, 11, and 13, plasmid DNA digested with *Swa*I after preincubation at 25, 30, 35, 40, 45 and 50°C, respectively, in the presence of BSA; lanes 4, 6, 8, 10, 12, and 14, plasmid DNA digested with *Swa*I after preincubation at 25, 30, 35, 40, 45 and 50°C, respectively, in the presence of recombinant *Hv*sHsp17.5-CI. SC, supercoiled plasmid; OC, open circular plasmid; L, linear plasmid. The numbers on the left represent the DNA markers in kb.

In order to obtain large quantity of highly purified *Hv*sHsp17.5-CI protein, a heterologous system was used to overexpress recombinant *Hv*sHsp17.5-CI protein in *E. coli*. The protein profiles of the *E. coli* BL21 (DE3) strain carrying the *Hv*sHsp17.5-CI-pET28a construct revealed overexpression of an approximately 20-kDa recombinant protein (Figure 8A), and most of it was located in the soluble fraction of the *E. coli* lysate. The recombinant *Hv*sHsp17.5-CI protein was purified to near-homogeneity (Figure 8A) from clarified *E. coli* lysate by Ni-NTA column chromatography. In contrast to Hsp60 and Hsp70 proteins, the chaperone activity of sHsps is ATP-independent [46]. The molecular chaperone activity of plant sHsps has been demonstrated both *in vitro* and *in vivo* [47]. *Swa* I restriction enzyme is thermo-labile and loses its enzymatic activity by pre-incubating at 37°C and above. We tested the protection of *Swa*I restriction enzyme (sensitive to high temperature) against thermal inactivation by pre-incubating 5 units of *Swa*I with 5 µg of either recombinant *Hv*sHsp17.5-CI or acetylated BSA at 25–50°C for 60 min before assaying the residual activity of *Swa*I on plasmid DNA. The restriction endonuclease *Swa*I completely lost its activity after 60 min of incubation at temperatures above 35°C. The pre-incubated *Swa*I was tested at 25°C for 60 min using supercoiled plasmid DNA (500 ng) containing a unique *Swa*I recognition site. DNA digestion profiles (Figure 8B) suggested that the recombinant protein and BSA were both able to protect *Swa*I activity up to 30°C (Figure 8B, lanes 6, 7). At temperatures above 35°C, BSA failed to protect against thermal inactivation of *Swa*I (Figure 8B, lanes 7, 9, 11, 13), whereas *Hv*sHsp17.5-CI provided significant protection (Figure 8B, lanes 6, 8, 10). Above 40°C, *Hv*sHsp17.5-CI provided only marginal protection. However, it was ineffective against thermal inactivation of *Swa*I at or above 45°C (Figure 8B, lanes 12, 14). These results suggest that the recombinant *Hv*sHsp17.5 keeps the *Swa* I restriction enzyme in folding competent state at higher temperature and the N-terminus hexahistidin tag is not interfering in this activity. The amount of the residual activity of *Swa* I restriction enzyme can be quantified by measuring the amount of DNA restricted.

## Discussion

The structure and function of the *sHsp* and *Hsf* family genes have been widely addressed in model plants like *Arabidopsis*, maize and rice [8], [13], [37]–[39], [48]. Most of the sHsps respond to a wide range of environmental stresses like heat, cold, drought, high light, salt, UV, oxidative stress and plant-pathogen interaction [5] and their concentration may even go up to 1% of the total proteins under high temperature stress. Proteins that are destabilized during cellular stresses are reactivated in the presence of ATP-dependent chaperones. We applied various bioinformatics approaches to analyze the phylogenetic relationship, conserved domains, localization prediction of proteins, *in silico* promoter analysis, transcript profiling and coexpression gene network analysis in barley to unravel its expression divergence and functional relevance of sHsps and Hsfs gene family members. Specialized *sHsp* and *Hsf* members which are preferentially expressed in specific organs/developmental stages (developing and imbibed seeds) and also influenced by drought stress conditions seem to suggest a conserved function of desiccation tolerance both during seed maturation events as well as during drought prone response in vegetative tissues.

The presence of HSE *cis* element is often correlated with the expression of respective *sHsp* and *Hsf* genes under heat stress as shown in the microarray analysis of *Arabidopsis*, rice and maize [5], [38], [39], [49]. The *cis*-motif ABRE [42] required for ABA response is present in the promoters of all the Hsf genes except in *HsfA2c*, suggesting that these *Hsf* genes are involved in ABA mediated signal transduction. The role of ABA in seed development [50], [51] and drought responses [52] is well known. Thus, ABA may be another important molecule in the regulation HSFs during seed development or drought stress. Within HsfA class, developmental expression of *HsfA9* in *Arabidopsis* is regulated by the seed specific transcription factor *ABSCISIC ACID-INSENSITIVE3* (*ABI3*). *ABI3* knock out lines lack *HsfA9* transcript and also seed abundant heat stress proteins like Hsp 17.4-CI, Hsp 17.7-CII and Hsp101 [15]. They concluded that *HsfA9* acts as a potent activator for the seed specific or developmental specific expression of Hsp genes in *Arabidopsis*. Recently, functional analysis of rice heat shock factor binding proteins *OsHSBP1* and *OsHSBP2* revealed their involvement in heat shock response [53]. Both these genes have been found important for seed development as their knockout lines are associated with significant seed abortion. Further research is required to clarify the expressions of sHsps and Hsf genes and their interplay during specific sexual processes.

Among *Hsfs*, *HsfA1a*, *HsfA2a*, *HsfB2c*, *HsfC2b* and *HsfA4b* were more preferentially expressed in endosperm and embryo (25 DAF) than others (Figure 2), suggesting a tighter coexpression with *sHsp17.7* (*CI* and *CII*), *17.5* (*CI*), and *19* (*CIII*). These specific gene family members of *sHsp* and *Hsf* genes are regulated by a defined developmental program such as embryogenesis and seed maturation events, a situation resembling with *Arabidopsis* and sunflower [15], [54]. Within this category, *HsfB2c* is identified as a putative central regulator represented in the vicinity of gene network of *Hv*Hsp CI, CII and CIII family members, and also the respective promoters are enriched with HSE *cis* element. Thus, *HsfB2c* is likely to mediate expression of these sHsp members. The analysis also identified the importance of *HsfB2c* that has not been previously implicated in plant stress responses and development and therefore *HsfB2c* might be one of the primary regulators of the HSR in barley. To prove the *in vivo* relevance of the *in silico* identified gene network, the molecular interaction of HvHsfB2c transcription factor on *HSE-box cis* element within the *HvsHsp17.5-CI* promoter was analysed. These results suggest that HvHsfB2c is responsible for the heat inducible transcriptional activation of *HvsHsp17.5-CI*. This transcriptional activation is mostly achieved by binding of HvHsfB2c to the HSE-box under heat stress conditions. It is rather surprising to note that typical feature of class A *Hsfs* which are known to possess transcriptional activator domain [55] remains unregulated under drought, while HsfB (*HvHsfB2b*, *HvHsfB2c*) and HsfC class (*HvHsfC1b*, *HvHsfC2b*) transcription factors were upregulated under drought in barley (Figure 3). While class *B-Hsfs* differ with class A not only in the lack of transcriptional activator AHA-motif, but also differ within oligomerization domain [56]. In this context, it is interesting to note the first emerging evidence we have shown in barley that HvHsfB2c as a central regulator mediates transcriptional response of *HvsHsp17.5-CI* gene, which is preferentially regulated during desiccation responses. Moreover, out of 5 reported class *B-Hsfs* in *Arabidopsis*, B3 and B4 are expressed at low level and HsfB1, HsfB2a and HsfB2b are significantly increased upon heat stress treatment [57], [58]. Similarly, the orthologue of *HsfB2C* in *Arabidopsis*, *AtHsf7* (AT4G11660) is expressed in developing seeds and found to be induced under stress. Also, the coexpressed genes depicted in the gene network of *AtHsf7* show embryo arrest in mutants and defective in thermo tolerance. These results suggest that *HvHsfB2c* in barley and *AtHsf7* are not only highly conserved in sequence and expression specificity between monocot and dicot lineages, but also seems to possess conserved function in seed development and stress tolerance. While none of the *HsfB* class members are characterized in monocots, studies from Ikeda et al. [59] indicate that HsfB factors suppress the general heat shock response under non-heat-stress conditions and in attenuating period they appear to be necessary for the expression of heat stress-inducible heat shock protein genes under heat stress conditions, which is necessary for acquired thermotolerance. The authors also mentioned that the heat stress response is finely regulated by activation and repression activities of Hsfs in *Arabidopsis*. Also, recent studies suggest that *Arabidopsis HsfB4* possess regulatory function in root development [60].

Based on coexpression network, it appears that HvsHsp 17.5 plays a vital role in barley seed development (Figure 4). Also, a strong interaction of a putative serine/threonine protein kinase, a calcyclin binding protein, ATP dependent RNA helicase and a metallothionein protein were observed in the gene network generated with that of sHsp17.5 (Figure 4 and Table S2). Serine/theronine protein kinase and calcyclin as a calcium binding protein acts as second messenger associated with the signal transduction. Metallothionins are not only associated with drought stress, but also involved in regulating zinc ion mobilization and metal homeostasis in late embryo developmental stages [61]. Cornet analysis emphasized the potential protein interaction partners as well the coexpressed gene regulatory networks of AT3G46230 (orthologous gene of *sHsp17.5* in barley) in *Arabidopsis*. These results suggest that AT3G46230 is likely to form protein complexes with several small heat shock proteins Hsp17.4, Hsp17.6 class I, Hsp17.6 class II, Hsp 17.6A and Hsp70 family (Figure 5). The presence of sHsps in different cell organelles indicate that potentially these genes might act as chaperones in protecting the various cellular compartments under stress as reported in case of mitochondrial Hsp22 of *Drosophila* [62].

Our results suggest that the *17.5CI sHsp* that we isolated from barley has chaperone activity and is able to protect the growth of *E. coli* and the activity of a thermolabile chaperone enzyme under heat stress. Based on our results, it is reasonable to speculate that the recombinant *Hv*sHsp17.5-CI protein might function as a molecular chaperone that conferred a moderate protective function against stress-induced protein damage in bacterial cells, as mostly these class of proteins might be involved in stabilization but not necessarily in refolding of denatured proteins under stress [3], [40]. This situation differs from high molecular weight Hsps which could refold denatured proteins under stress but requires ATP [33]. Cytosolic Hsp17.7 and Hsp17.3 of tomato have been shown to act as molecular chaperones *in vivo* [63] and also an overexpression of *AtHSP17.6A* lead to enhance osmotolerance [64]. Chauhan et al. [49] showed that a chloroplastic *TasHSP26* is involved in seed maturation and germination and its heterologous expression results in tolerance to heat stress in *Arabidopsis*. Thus, it appears that some of these plant specific sHsps act not only as molecular chaperones under stress, but also likely to possess similar function in developmental programs especially in the seeds.

## Supporting Information

Figure S1 **Multiple sequence alignment of the Hsf protein family in barley with corresponding members of rice.** Different classes of the HSF numbers correspond to the order of the alignment. The multiple alignment results clearly show the highly conserved DBD domains among all the Hsf genes which are marked with dotted boxes. The secondary structure elements of DBD (α1-β1- β2-a2-a3- β3-β4) are shown above the alignment. These were predicted based on the PSIPRED protein structure prediction server. The scheme at the top depicts the locations and boundaries of the HR-A core, insert and HR-B regions within the HR-A/B regions which are marked with thick boxes. Positions of the other identified motifs nuclear localization signals (NLS) are highlighted with yellow colour, nuclear export signal (NES) highlighted in yellow colour with underline and activator (AHA) motif sequences are shown in the red colour and each motif name is mentioned above the alignment. Alignment was performed by using ClustalW (DNAstar) program.
(PDF)

Figure S2 **Multiple alignment of different sub-classes of** ***Hv*** **sHsps.** The conserved α- crystallin domain was labeled with dotted box. The defined consensus regions I and II are marked with underline below the sequences. Highly conserved and semi-conserved regions are shown in “*” and “.”, respectively. Small Hsp region specific to respective subclasses was labeled with thick boxes. The chloroplast localized proteins have transit sequences that are specific for organelle and is labeled with red colour. The conserved Arg is displayed in red colour in the β7 strand. The secondary structure assignments for all classes of *Hv*sHsps were labeled above the sequences. The predicted β-strands depicted by thick lines above the alignment are based on their position in known secondary structure of Hsp16.9 from *T. aestivum* (van Montfort et al. 2001). The IXI/V motif in the C-terminal extension is shown in green. The SXXFD motif and interacting residues in the conserved alpha crystallin domain are in pink colour. Alignment was performed by using ClustalW (DNAstar) program.
(PDF)

Figure S3 **Position of putative** ***cis*** **-elements present in the promoter regions of barley Hsf genes.** The analysis was performed using PlantCARE and PLACE databases. The “rectangle mark” shows the relative position of the different motifs.
(PDF)

Figure S4 **Position of putative** ***cis*** **-elements present in the promoter regions of barley sHsp genes.** The analysis was performed using PlantCARE and PLACE databases. The “rectangle mark” shows the relative position of the different motifs.
(PDF)

Figure S5 **Subcellular localisation of HvHsfB2c in transient transformed Arabidopsis thaliana mesophyll protoplasts for the the N-terminal CFP-fusion derivatives.** scale = 10 µM. A, N-terminal CFP-fusion; B, C-terminal CFP fusion.
(PDF)

Table S1 **Barley sHsp and Hsf Orthologous genes in** ***Arabidopsis*** **.**
(PDF)

Table S2 **Additional file 5: Detailed list of functional annotations of genes represented in the network of** ***HvHsfB2c/HvHsp17.5-CI*** **.**
(PDF)
