# Supplementary material for: Unraveling Regulation of the Small Heat Shock Proteins by the Heat Shock Factor HvHsfB2c in Barley: Its Implications in Drought Stress Response and Seed Development
Source: PLoS One. 2014 Mar 4;9(3):e89125. doi: 10.1371/journal.pone.0089125 (PMC3942355; doi:10.1371/journal.pone.0089125)
Supplement: Figure S1 — Multiple sequence alignment of the Hsf protein family in barley with corresponding members of rice. Different classes of the HSF numbers correspond to the order of the alignment. The multiple alignment results clearly show the highly conserved DBD domains among all the Hsf genes which are marked with dotted boxes. The secondary structure elements of DBD (α1-β1- β2-a2-a3- β3-β4) are shown above the alignment. These were predicted based on the PSIPRED protein structure prediction server. The scheme at the top depicts the locations and boundaries of the HR-A core, insert and HR-B regions within the HR-A/B regions which are marked with thick boxes. Positions of the other identified motifs nuclear localization signals (NLS) are highlighted with yellow colour, nuclear export signal (NES) highlighted in yellow colour with underline and activator (AHA) motif sequences are shown in the red colour and each motif name is mentioned above the alignment. Alignment was performed by using ClustalW (DNAstar) program. (PDF) [file pone.0089125.s001.pdf]

Figure S1

a)

```
                                                                                             α1                β1         β2       α2                                               α3            β3
HvHsfA1a MEGGVALASS----VTTAVAPP--------------------------GQGAG--------------------APP---PFLMKTYDMVDDPATDAVVSWG-PASNSFIVWNTPEFARDLLPKYFKH-----------------NNFSS---FVRQLNTYGFRKVD   92
OsHsfA1a MEAAVAAAAAAAGAVTTAVAPP-------------------------PGAAVSNG---------------VATAPP---PFLMKTYEMVDDPATDAVVSWG-PGNNSFVVWNTPEFARDLLPKYFKH-----------------SNFSS---FVRQLNTYGFRKVD  102
HvHsfA2a ------MDPFH---GIVKEEEFDFAGAAADGYSPSSWGSSPSSWGSSQSSWVGGGA------LAELPRPMDGLGEAGPTPFLNKTYEVVDDHSTDTIVSWG-VAGNSFVVWDAHAFSMVLLPRYFKH-----------------SNFSS---FVRQLNTYGFRKVD  130
OsHsfA2a ------MNPLR---VIVKEEELDFAAAAAAAAA------GE--G--SPSSWAVG--------VMDLPRPMEGLGEAGPPPFLCKTYEVVDDPGTDTVISWG-FAGNSFVVWDANAFAAVLLPRYFKH-----------------SNFSS---FVRQLNTYGFRKVD  118
HvHsfA2b ------MEDPVP--SLVKEEEE-----EG--------AHGR------GGSPGVGA----------APRPMEGLHDAGPPPFLTKTYDMVDDPNTDSVVSWS-AGNNSFVVWDPHAFATVLLPRHFKH-----------------SNFSS---FVRQLNTYGFRKVD  108
OsHsfA2b ------MDDPML--NAVKEEES-----HGDGGGLEV-VAGE------DGAAAVAA--------GVAPRPMEGLHDAGPPPFLTKTYDMVDDAGTDAAVSWS-ATSNSFVVWDPHAFATVLLPRFFKH-----------------NNFSS---FVRQLNTYGFRKVD  117
HvHsfA2c ------MDAAVP--DGIVKEEEGLLLLHEERPPAAG-PPQR------QRQEEDGA----------LPRPMEGLHEAGPPPFLTKTYDLVEDPATDQVVSWS-RAGNSFVVWDPHVFADALLPRLFKH-----------------SNFSS---FVRQLNTYGFRKVD  120
OsHsfA2c ------MDPAA---AGIVKEE----MLESQ---------QQ------QRQEDGGA----------APRPMEGLHEVGPPPFLTKTYDLVEDPATDGVVSWS-RAGNSFVVWDPHVFADLLLPRLFKH-----------------NNFSS---FVRQLNTYGFRKVD  107
HvHsfA2d -------------------------------------------------------------------------------------------------------------------------------SNYAD----LFCN-FQGFRKID   17
OsHsfA2d ------MEKMM---PGMVKEE------------------WP------PSSPEEGE----------APRPMEGLHEVGPPPFLTKTFDLVADPATDGVVSWG-RAGSSFVVWDPHVFAAVFLPRFFKHNNFSSFVRQLNTYFLVRTNYLNKRSHFYSLRFQGFRKID  122
HvHsfA2e MGSAAAMSHRMM--NPVKVEGR-----PSP-------SPSP------TAAAGGGG--------G-APRPMDGLADAGPTPFLAKTYDMVDDPTTDAVVSWS-ATNNSFVVWDPHLFGTVLLPRYFKH-----------------NNFSS---FVRQLNTYGFRKVD  116
OsHsfA2e ------MNYRVV--NPVKVESG-----PS------------------TGVANGQ-----------PPRPMDGLADGGPPPFLTKTYDMVDDPTTDAVVSWS-ATNNSFVVWDPHLFGNVLLPRYFKH-----------------NNFSS---FVRQLNTYGFRKVD  103
HvHsfA2f ------MDSAAAMTMDIKQEQPEMMVLD-----------DD------DDDAGCCL----------APTPLDLAAAAAVAPFLAKTFDMVEDPATDAVVSWG-AARNSFVVWDPHAFAAGLLPLHFKH-----------------ANFSS---FLRQLNTYGFRKVN  112
OsHsfA2f ------MDYS-----TVKQEEVEVVVLDG----------EE------EAAAAAAP----------VPLPAAMGVGAAVAPFLVKTFEMVEDPATDAVVSWGGAARNSFVVWDPHAFAAGLLPLHFKH-----------------ANFSS---FLRQLNTYGFRKVS  109
HvHsfA4b ----------------ME----------------------------------------------------GSGGSSSLPPFLIKTYEMVDEPATDAVVAWTPS-GTSFVVFSQADFCRDLLPKYFKH-----------------NNFSS---FVRQLNTYGFRKVD   77
OsHsfA4b ----------------ME----------------------------------------------------GGGGGGSLPPFLSKTYEMVDDPSTDAVVGWTPA-GTSFVVANQPEFCRDLLPKYFKH-----------------NNFSS---FVRQLNTYGFRKVD   77
HvHsfA4d ----------------MEME--------------------------------------------------GGSQGASPAPFLIKTYEMVEDPATSRVVSWGPG-GASFVVWNPPDFSRDLLPKYFKH-----------------NNFSS---FIRQLNTYGFRKID   79
OsHsfA4d ----------------MESS-------------------NL---------G-GGG---------------GGGGGGGPPPFLIKTYEMVEDAATNHVVSWGPG-GASFVVWNPLDFSRDLLPKYFKH-----------------NNFSS---FIRQLNTYGFRKID   85
HvHsfA5  ----------------MEAS-------------------GA---------A--GA---------------RGGGGGGPAPFLLKTYEMVDDPATDAVVSWSDASDASFVVWNSPEFAARLLPAYFKH-----------------SNFSS---FIRQLNTYGFRKID   85
OsHsfA5  ----------------MEVA-------------------AG---------ARGGG---------------AGGGGGGPAPFLLKTYEMVDDPSTDAVVSWSDASDASFVVWNHPEFAARLLPAYFKH-----------------SNFSS---FIRQLNTYGFRKID   87
HvHsfA9  ---------------SARGAP----------------------------VG-------------------AVPKPPXVAPFLTKVYDMVSDAATDRVMSWS-DAGNSFVIWDAHAFERDLLRHHFKH-----------------NNFSS---FIRQLNTYGFRKVD   83
OsHsfA9  MGSKKRSPQH-----PAAAAPP-------------------------PAVGGGGGGEVSGDGGASTANGPVVPKPSEVAPFLTKVYDMVSDPATDNVISWA-EGGGSFVIWDSHAFERDLHR-HFKH-----------------SNFTS---FIRQLNTYGFRKVH  114
                                                                                                                           DBD

              β4                                                                                      HR-A-Core              Insert              HR_B                      NLS
HvHsfA1a PDKWEFANEGFLRGQKHLLKTINRRKPLH-------ANNQVQVQQQQHQQQHQQQPQLQNAPIPSCVEVGK-FG-MEEEIEMLKRDKNVLMQELVRLRQQQQTTDHQLQTLGKRLHGMEQRQQQMMSFLAKAMQSPGFLAQFVQQNENSK--RR-----IVAANKK  242
OsHsfA1a PDRWEFANEGFLRGQKHLLKTINRRKPTH-------GNNQVQ------------QPQLPAAPVPACVEVGK-FG-MEEEIEMLKRDKNVLMQELVRLRQQQQTTDHQLQTLGKRLQGMEQRQQQMMSFLAKAMHSPGFLAQFVQQNENSR--RR-----IVASNKK  240
HvHsfA2a PDRWEFAAEGFLRGQKELLKTIRRRRP--------QSSGTPG------------QQQQQQGG--VCLEVGH-FG-HDGEVQQLKRDKGTLIAEVVKLRQEQQATRVQMQAMEARLAATEQKQQQMTVFLARAMKSPSFLQMLVERQD--QSRRKELADALLS-KKR  269
OsHsfA2a PDRWEFANEGFLRGKKELLKTIKRRRPPPSSPPSSSSSSSSS------------QHQQQPAA--ACLEVGQ-FG-RDGVVNRLQRDKSVLIAEVVKLRQEQQTTRAQMQAMEERISAAEQKQQQMTVFLARAMKNPGFLQMLVDRQAGQHGARNRVLEDALS-KKR  267
HvHsfA2b PDRWEFANEGFLRGQRHLLKNIRRRKP----------PAHTA------------SNQQ-SLG--SYLEVGH-FG-NDAEIDRLKRDKQLLMAEVVKLRQEQQNTKAHLKAMEDRLRGTEQKQQQMTSFMARVLRNPEFLKQLISRNG-----MRKELHEAIS-KKR  241
OsHsfA2b PDRWEFANENFLRGQRHLLKNIKRRKP----------PSHTA------------SNQQ-SLG--PYLEVGH-FG-YDAEIDRLKRDKQLLMAEVVKLRQEQQNTKANLKAMEDRLQGTEQRQQQMMAFLARVMKNPEFLKQLMSQNE-----MRKELQDAIS-KKR  250
HvHsfA2c PDRWEFANEGFLRGQRHLLKMIKRRKP----------PSNAP------------PSQQQALT--SCLEVGE-FG-FEEEIDRLKRDKNLLITEVVKLRQEQQATKDNVQAMENRLQAAEQKQAQMMGFLARAMRNPLFFQQLVQRQD-----KRKELEDAIS-KKR  254
OsHsfA2c PDRWEFANEGFLRGQRHLLKTIKRRKP----------PSNAP------------PSQQQSLT--SCLEVGE-FG-FEEEIDRLKRDKNILITEVVKLRQEQQATKDHVKAMEDRLRAAEQKQVQMMGFLARAMRNPEFFQQLAQQKE-----KRKELEDAIS-KKR  241
HvHsfA2d PDRWEFANEGFIRGQRQLLKMIKRRRP----------LSYLP------------SSQQQALG--SCLEVGQ-FG-FDDEIEVLKRDKNALLSEVVKLRQEQQSTRADMRAMEERLHRAEQKQLQMMGFLARAIQNPDFFLQLVQQQD-----KLKDLEDPYP-TKR  151
OsHsfA2d PDRWEFANDGFLRGQRHLLKMIKRRRP----------LSYLP------------GSQQ-ALG--TCLEVGQ-FG-LDEEIDRLKRDKNILLAEVVKLRHKQQSTKANMRAMEERLQHAEQKQVQMMGFLARAMQNPDFFHQLIHQQD-----KMKGLEDTFS-KKR  255
HvHsfA2e PDRWEFANEGFLRGQRHLLRNIKRRKP----------T-HGS------------QNQQ-SLG--SYLEVGN-FG-HDVEIDQLKRDKQLLMAEVVKLRQEQQNTRSDLQAMEKRLQGTEQKQQQMMSFLARVMQNPLFIRQLISQSE-----MRKELEDAIS-NKR  248
OsHsfA2e PDKWEFANEGFLRGQKHLLKSIKRRKP----------P-NSS------------PSQQ-SLG--SFLEVGH-FG-YEGEIDQLKRDKHLLMAEVVKLRQEQQNTKSDLQAMEQKLQGTEQKQQHMMAFLSRVMHNPEFIRQLFSQSE-----MRKELEEFVS-KKR  235
HvHsfA2f PDRWEFANAGFLGGQRHLLAGIRRRR-----------GADTG------------RRPAAALSPSSCAEGAGGFGSVEGELERLRQDREALKRELAGLKRQQVEARATLLDMERRVEDTERRQEQCKAFLARAVRNPAFLANLARRND----LAAAAPAPAVDGKKK  251
OsHsfA2f ADRWEFANEDFLGGQRHLLANIRRRRR----------GAGTG------------STTPRAVN---CGGGGG-----EGEVERLRRDKEALARELARLRRQQQEARAQLLDMERRVRGTERRQEQCTEFLARALRSPDVLDNIARRH---------AAA--VE--RK  232
HvHsfA4b PEQWEFANEEFIRDQRHRLKNIHRRKP----------IFSHS------------SHT-QGAGPLADSERRD----YEEEIERLKCENASLNLQLER---KKTDMDSKMKALEDKLLAIEDQQRNLISYVTEIVKAPG--FLSS------------FIEQSDHHGKK  199
OsHsfA4b PEQWEFANEDFIKGQRHRLKNIHRRKP----------IFSHS------------SHS-QGAGPLTDNERKD----YEEEIERLKSDNAALSSELQNNTLKKLNMEKRMQALEEKLFVVEDQQRSLISYVREIVKAPG--FLSS------------FVQQQDHHRKK  202
HvHsfA4d PERWEFANDDFIRGHMHLLKNIHRRKP----------VHSHS------------LQN-QVNGPLAESERRE----YEDEISRLKHENSLLVAELQKQAHQQCGIGWLMQSLEDRLMVMEQRQTDVVSSVRDILQRRRGAHHPGQQTM-------LELEPTDRFSKK  211
OsHsfA4d PERWEFANEDFIRGHTHLLKNIHRRKP----------VHSHS------------LQN-QINGPLAESERRE----LEEEINRLKYEKSILVADLQRQNQQQYVINWQMQAMEGRLVAMEQRQKNIVASLCEMLQRRGGAVSSS-------------LLESDHFSKK  211
HvHsfA5  PERWEFGNEYFVKGQKHLLKNIYRRKP----------IHSHS------------HQ----PAAQSDNERSF----FEDEIDRLAREKANLQAELWKFKQQEPGTMFQIEALERRAVDMEQRQGKMIAFLQQASKNPHFVSKLVKMAE-------ASSMFADALHKK  214
OsHsfA5  PERWEFANEYFIKGQKHLLKNIHRRKP----------IHSHS------------HP----PGALPDNERAI----FEDEIERLSREKSNLQADLWKSKQQQSGTMNQIEDLERRVLGMEQRQTKMIAFLQQASKNPQFVNKLVKMAE-------ASSIFTDAFNKK  216
HvHsfA9  PDRWEWANEGFLRGQKHLLKIIKRKKRP--------QEASRE----------LEKAPVKASPGTENIEIGR-YGGLVKEVETLKRDKALLMQQLVDLRHYQQSSNLEVQSLIQRLQLMEQNQKQMMALLAIVVQNPSLLNQLVQQQQQQQ--RRNSWR-YEDGNKK  227
OsHsfA9  PDRWEWANEGFIMGQKHLLKTIKRRKKSS-------QESPSE----------IQKAPVKTAPGTENIEIGK-YGGLEKEVETLKRDKALLMQQLVDLRHYQQTSNLEVQNLIERLQVMEQNQQQMMALLAIVVQNPSFLNQLVQQQQQQ---RRSNWW-SPDGSKK  258
```

```
HvHsfA1a RRLPKQDDG--LN------PESALLDGQIIKYQPMINEAAKAMLRKILQQDTSPHRFESMGNSDNLLLENCMPSAQTFDSSSSTRNSA-----------VTLAEVPGNS---------GMPYMPTSSGL-------SAICSSSSPPEMQCPPVLD----------  362
OsHsfA1a RRLPKQDGS--LD------SESASLDGQIVKYQPMINEAAKAMLRKILKLDSS-HRFESMGNSDNFLLENYMPNGQGLDSSSSTRNSG-----------VTLAEVPANS---------GLPYVATSSGL-------SAICSTSTP-QIQCPVVLD----------  358
HvHsfA2a GRPIEYLLPR-NG------ETCYS--AAAQGYGHGL--------------------VD---------------------------------------------------------------------------GGEGRRADGEDTESFWK----------  315
OsHsfA2a RRPIEYLLTR-NG------ETCAAGESAAMLAADGV--------------------AEPDG-------------------------------------------------------DT-------TPRGDGGGGGGGDTESFWM----------  322
HvHsfA2b RRRIDGGPE--AY------DVGAS-SSSLEQESPVV--------------------FDSHGSVELLADVSVPVELLADGIPPDLEGSV-----------ELLADGIPPDLEGSVALLADGIPPDLEGSV-------ELLVDG-IPSDLGGSGIDA----------  348
OsHsfA2b RRRIDQGPE--VD------DVGTS-SS-IEQESPAL--------------------FDPQ-----------------------------------------------E--------SV-------EFLIDG-IPSDLENSAMDA----------  301
HvHsfA2c RRPIDNVPFY-GS------GVTTS-QS-EQLDSQFL--------------------FDSG---------------------------------------------------------VL-----------SDPGTHRLENLAQNI----------  302
OsHsfA2c RRPIDNVPFY-DP------GE-TS-QT-EQLDSPYL--------------------FDSG---------------------------------------------------------VL-------NEL-SEPGIPELENLAVNI----------  291
HvHsfA2d RRSINVMPFL-GP------EG-TS-QS-EQLESTFI--------------------FEDR---------------------------------------------------------EF----------------SELENLAMNI----------  193
OsHsfA2d TRSIDIVPFL-NP------GE-VS-QG-DQLESTLL--------------------FDPR---------------------------------------------------------PF-------AELNDEPAKSELENLALNI----------  306
HvHsfA2e RRRIDQGPEA-VD------SMGTG-ST-LEQGSHVM--------------------FEQQ-----------------------------------------------E--------PV-------DSFVNG-VISDLESSSVDT----------  300
OsHsfA2e RRRIDQGPE--LD------SMGTG-SS-PEQVSQVM--------------------FEPH-----------------------------------------------D--------PV-------DSLFNG-VPSDLESSSVEA----------  286
HvHsfA2f RRRLDAIPSPLPA------EDGFTFEE-LALAAGVV--------------------EEA-------------------------------------------------------------------AAPTQGSGAGGVTTDMIWY----------  302
OsHsfA2f KRRMLAAAAD---------DDGLTDFEA-LALAA--------------------------------------------------------------------------------------------AADTSHSTGGAVTTDMIWY----------  274
HvHsfA4b RRLPKSISFHEGAS-----TQGNQIMHCDLANSPAHKLYR-----------ESFDKMESSLNSLENFFKEAT-EALGNDISYDGDVPR---HSSAVVLTELHSSGESDPHAQSPPSMMHTCSAGVGDSHS-------SRDIAESASC-PESPPLPEAHSRADSRAK  337
OsHsfA4b RRLPIPISFHEDAN-----TQENQIMPCDLTNSPAQTFYR-----------ESFDKMESSLNSLENFLREAS-EEFGNDISYDDGVPG---PSSTVVLTELHSPGESDPRVSSPPTRMRTSSAGAGDSHS-------SRDVAESTSC-AESPPIPQMHSRVDTRAK  340
HvHsfA4d RRVPKIDLFVE--------EQRVPYPRAIGDETPGMIQVN----------AEPFEKMEMALVSLEKLVQRA--AAATPTPSTDDPALG-----------DLQAAPMEAGVNLEL------SPPNIRHVHSPP-----ELAVAESPGYAVQSPMLLFPDIQQDKHKT  335
OsHsfA4d RRVPKMDLFVDDCAAG--EEQKVFQFQGIGTDAPAMPPVLP------VTNGEAFDRVELSLVSLEKLFQRAN-DACTAAEEMYSHGHGGTEPSTAICPEEMNTAPMETGIDLQLPASLHPSSPNTGNAHLHL-----STELTESPGF-VQSPELPMAEIREDIHVT  362
HvHsfA5  RRLSGLDYAVEATETASFCDDHSATSRQEMGNLLNQHFSDK------LKLGLCPAETESNLITLSTQSSHEDNCSLHGRHPPGHDGMGTGCLPLVPQMMELSDTGTSICPSKSSFFRPAVNDEGLSPCHLSLTLASCSMDVDRGQASNADGSTTVN--KGSDNPLE  372
OsHsfA5  RRLPGLDYSIENTETTSFYDDHSSTSKQETGNLLNQHFSDK------LRLGLCPAMTESNIITLSTQSSNEDNRSPHGKHP-ECDMMGRECLPLVPQMMELSDTGTSICPSKSSCFAPPISDEGLLTCHLSLTLASCSMDVDKSQGLNANGTTIDNPTEAATATME  375
HvHsfA9  RRFPALEQGPVTD------QETSGAGAEIIQYRPPV------------P--------ETS--------------------S-----Q------------VIADE----------------------------------AYLSATAE-PISSPPLN-----------  284
OsHsfA9  RRFHALEQGPVTD------QETSGRGAHIVEYLPPV------------P--------ETS--------------------G-----Q------------VNPVEG---------------------------------AICSANSQ-PVPSPAVA-----------  316
```

```
                                                   AHA-motif                                                                                                                             NES
HvHsfA1a ----------SNSSTQLPNMSAVPSVPKA---MTPGLSD-----ISIPGFPDLHDLITEDAINIPVENYAMPGPECIFPLPEGSDDSVPMDPIDTDEIDDTQKLPGIIDSFWEQFLCASPLSVDNDEVDSGLLDTREAQEENGWTRTENLANLTEQMGLLSSNHRG  510
OsHsfA1a ----------NGIPKEVPNMSAVPSVPKA---VAPGPTD-----INILEFPDLQDIVAEENVDIPGGGFEMPGPEGVFSLPEEGDDSVPIETDEILYNDDTQKLPAIIDSFWEQFLVASPLSVDNDEVDSGVLDQKETQQGNGWTKAENMANLTEQMGLLSSHHTG  506
HvHsfA2a ----------ELLSLGLEER-----QREA---GGGGGGE-----AS---GAEVD-----------------------------------------DDVD-----------------------------------DEMDELVQSLYHLSPNRPHGHHHQ  371
OsHsfA2a ----------QLLSLGLEEK-----QRED---GVAGGVQ-----ESNSGGADVDND---E-----------------------------------------EDDD-----------------------------------DDVDVLVQSIYHLSPK  376
HvHsfA2b ----------NGV-TEPQDFGLGTCEPQQ---NRVP-GL-----FRDSFWEELLNKGLS-----------------------------------------DESD-------------------------------EPVNVDGMDVLSEKMGYFIPNSPTQTT  418
OsHsfA2b ----------GGL-VEPQDFDVGASEQQQ---IGPQ-GE-----LNDNFWEELLNEGLVG-----------------------------------------EEND-------------------------------NPVVEDDMNVLSEKMGYLNSNGPTAGE  372
HvHsfA2c ----------QDLGQGKTDE-----GKKD---EANGQVE-----LNNEFWAELFSDDFGD-----------------------------------------GDGSGLSE--------------------------LEGRRPEDIDELAQQLGYLSSTSPL  372
OsHsfA2c ----------QDLGKGKVDE-----ERQN---QTNGQAE-----LGDDFWAELLVEDFTG-----------------------------------------KEEQ--SE--------------------------LDG-KIDGIDELAQQLGYLSSTSPK  358
HvHsfA2d ----------QGIRKGMEDD----KGGRS---QGCGEAE-----LTDDFWEELLSEGMRD-----------------------------------------EAEM--LE--------------------------LERRRSRYVDA  248
OsHsfA2d ----------QGLGKGKQDVNRTRNQPRN---QASNETE-----LTDDFWEELLNEGARD-----------------------------------------DAGI--PG--------------------------MERRRPRYVDALAQKLGYLSNSSQK  379
HvHsfA2e ----------KGA-EVQQSVASSRSEQLR---GRPS-GE-----LNDDFWEDLLHEGGLG-----------------------------------------EEAS-------------------------------NLVVPDDMNLLAQEMH  360
OsHsfA2e ----------NGG-KAQQDVASSSSEHGK---IKPSNGE-----LNEDFWEDLLHEGGLD-----------------------------------------EDTR-------------------------------NPAI-DDMNLLSQKMGYLNSSSTKSPQ  357
HvHsfA2f ----------ELLEEGQAEID---VEVED---LVAAAG------------DMAP---WE-----------------------------------------FGEE-----------------------------------EDPDDGVCRDARRGRE  350
OsHsfA2f ----------ELLGEEQAEID---IEVDQ---LVASASA-----AADT-ASEAEP---WE-----------------------------------------EMGE-----------------------------------EEVQELVQQIDCLASPSS  331
HvHsfA4b ---------VSDIDVNLEPAVTETGPSRDQQPTQDPPAD-----ANDGFWQQFLTEQPGS----------------------------------SDTHQEAQSERRD------------REANQTVTRDRGSFWWG-KSVEQMTEKLGHLTSAEKT  432
OsHsfA4b ---------VSEIDVNSEPAVTETGPSRDQ-PAEEPPAVTPG--ANDGFWQQFLTEQPGS----------------------------------SDAHQEAQSERRDG-----------GNKVDEMKSGDRQHLWWGKRNVEQITEKLGLLTSTEKT  440
HvHsfA4d ---------MSEADLSSEAS--TTDTSQDETTAETGVPREPAVAANDLFWERFLVDTPKP----------------------------------QCGYAFQESHESK---------------DDVKIGIDCNWYGHRDNVDQITQQMGHLASAQKT  431
OsHsfA4d RY-------PTQADVNSEIAS-STDTSQD-GTSETEASHGP---TNDVFWERFLTETPR-----------------------------------SCLDESERQESPK---------------DDVKAELGCNGFHHREKVDQITEQMGHLASAEQTLHT  459
HvHsfA5  AT---AAAMGSNQKASVDAG-TDATTPREDTRVATEPAAP--VKANDNFWEQFLTERPG----------------------------------DASSGPRDP---------------S----MDKGQTEGREKDDREATEQLKL  457
OsHsfA5  KDDTIDRSFDDNQKKSADSRTADATTPRADARVASEAPAAPAAVVNDKFWEQFLTERPGC---------------------------------SETEEASSGLRTD---------------TSREQMENRQAYDHSRNDREDVEQLKL  475
HvHsfA9  ------------MPMDIDTETTSDNL------NTQGSSG-----DIFADMPALPDFDD-----------M----Q---LWFGE--------------DGEL----------------TLTIQD-------YDESPQSEQDCQMEAQHNHNNPQYASR  363
OsHsfA9  ------------TPMDMQTSNVAD---------TLGSSE-----EPFADNSTLHEWDDND----------M----Q---LLFDDN----LDPILPPFENDGQMGP-------------PLSVQD-------YD-FPQLEQDCLMEAQYNSNNPQYADVITEA  410
```

b)

```
                                                                            α1            β1        β2     α2                 α3       β3
HvHsfB1   --VIIKRAGMAGAAAAAQQQQKGGGGV--------------V--------MKGG---GGGPAPFLTKTHQMVEERGTDEVISWGEH-GRSFVVWKPVELARDLLPLHFKHCNFSSFVRQLNTYGFRKVVPDR      104
OsHsfB1   ------MAAAEAAAAVGKQQQKGGGG------------------------RGGG---GGGPAPFLTKTNQMVEESATDEVISWGKE-GRSFVVWKPVEFARDLLPLHFKHCNFSSFVRQLNTYGFRKVVPDR       98
HvHsfB2a  ------------MASPAL----GAG-----------------------------------TPPFLTKTYAMVDDPETDDTISWNES-GTAFVVWRRAEFERDLLPKNFKHSNFASFVRQLNTYGFRKIGLDR       80
OsHsfB2a  ------------MASPA------AG-----------------------------------TPPFLTKTYAMVEDPSTDETISWNDS-GTAFVVWRPAEFARDLLPKHFKHSNFSSFVRQLNTYGFKKVVADR       78
HvHsfB2b  -------MVSSGQICTVV----GGGG--VMGEQAAPVSCGETPAQAEVGTGVGQ---RSLPTPFLNKTYQLVDDPAVDDVISWSED-GSAFVVWRPAEFARDLLPKYFKHNNFSSFVRQLNTYGFRKIVPDR      115
OsHsfB2b  -------MADQTAAAVVV----GGGAAATMGEPSPPPPA-PAAEAAGVGVGQQQ---RTVPTPFLTKTYQLVDDPAVDDVISWNDD-GSTFVVWRPAEFARDLLPKYFKHNNFSSFVRQLNTYGFRKIVPDR      116
HvHsfB2c  ------MAAEHGAAAAAV----DGAG-------AEPPPPAPMPGLAATADAAGQ---RSLPTPFLTKTYQLVEDPAVDDVISWGED-GSTFVVWRPAEFARDLLPKYFKHNNFSSFVRQLNTYGFRKIVPDR      111
OsHsfB2c  -------MAEQGAGEADA----GGG---------EPPPAAVMT--AAAEALAGQ---RSLPTPFLTKTYQLVEDPAVDDVISWNED-GSTFVVWRPAEFARDLLPKYFKHNNFSSFVRQLNTYGFRKIVPDR      106
HvHsfB4b  MAFLVERCGEMVVSMEMGSGAHGGGG-----------------------GGGGGGVGKPVPAPFLTKTYQLVDDPCTDHIVSWGED-DATFVVWRPPEFARDLLPNYFKHNNFSSFVRQLNTYGFRKIVADR      108
OsHsfB4b  MAFLVERCGEMVVSMEMGP--HGGGG-----------------------AAAG----KPVPAPFLTKTYQLVDDPCTDHIVSWGED-DTTFVVWRPPEFARDLLPNYFKHNNFSSFVRQLNTYGFRKIVADR      102
HvHsfB4c  ----MERCGSWSESDAAAQ------------------------------AAAQ----KAVPAPFLTKTYQLVDDPATDHIVSWGDDRVSTFVVWRPPEFARDILPNYFKHNNFSSFVRQLNTYGFRKVVPER       94
OsHsfB4c  ----MERCGSWSDCEAAA-------------------------------AAAQ----KAVPAPFLTKTYQLVDDPATDHIVSWGDDRVSTFVVWRPPEFARDILPNYFKHNNFSSFVRQLNTYGFRKVVPER       93

            β4
HvHsfB1   WEFANENFRRGEQSLLSGIRRRKATTT-----------------TTTPQSSKTCGTAVN--------------------VAFP-------PP---LPALPP-------ASASTSGTGNDLSSS---------      173
OsHsfB1   WEFANGNFRRGEQGLLSGIRRRKATT---------------------PQSSKSCGSGVN--------------------VAFP-------PP---LPPLPP-------EPSATTSSGNDRSSS---------      163
HvHsfB2a  WEFANECFRKGEKQLLGAIQRRKGSG-------------------AGAPAPAMMATPIA--------------------TAIPISP----TP--TSSGGDP-------AVSSSPPPGLALVAT---------      151
OsHsfB2a  WEFANDCFRRGEKHLLGGIQRRKGSG-------------------TGG-AGAAPAGGIP--------------------TAIPISS----PP--TSSGGEP-------AVSSSPPRGAAGIAAGV-------      150
HvHsfB2b  WEFANDCFRRGEKRLLCDIHRRKVTPTV----------------AATAAVTVAAAAAIP--------------------VALPVTK-RQGSP--VLSGDEQ-------VLSSSSSPEPPFLNQYAPSY----      197
OsHsfB2b  WEFANDCFRRGERRLLCEIHRRKVTP------------------PAPAATTAAVAAAIP--------------------MALPVTTTRDGSP--VLSGEEQ-------VISSSSSPEPPLVLPQAPSG----      197
HvHsfB2c  WEFANDCFRRGEKRLLCDIHRRKVVQSA---------GLAAAAAAAAAGAVTVAAAAIP--------------------MALPVTR--SGSPELQLSSEEQ-------VLSSNSGSAEELPLAPSGSGGSAP      205
OsHsfB2c  WEFANDCFRRGEKRLLCDIHRRKVVAAAAAAPPPPSPGMATAAAAVASGAVTVAAAPIP--------------------MALPVTR--AGSP--AHSSEEQ-------VLSSNSGSGEEHRQA-SGSG-SAP      205
HvHsfB4b  WEFANEFFRKGAKHLLAEIHRRKSS----------------------QPPPPSMVPHQAAYH-------HHHYHLG---NTFSPQP---PPPAAAAHHQHHP------VYQQQHFVQEEPAAAAA-------      192
OsHsfB4b  WEFANEFFRKGAKHLLAEIHRRKSS----------------------QPPPP-PMPHQP--Y-------HHHHHL----NPFSLPP---PPP---AYHHHH-------LIQEEPATTAHCTVAGD-------      178
HvHsfB4c  WEFANEFFRKGEKQLLCEIHRRKTSGST--------------T-SS-SPPPFFAPPHFPLFH-PGVSVAHHHQQFVGEDGAMAAHAG-MGVPFMQPHWREQ---GAPRLLALGGPAAPSPGVE---------      196
OsHsfB4c  WEFANEFFRKGEKQLLTEIHRRKTSSAS--------------T-ASPSPPPFFAPPHFPLFHHPGVAAAQHHHAFVGDDGVVAAHG--IGMPFPQPHWREPNLPVATRLLALGGPAPSPSSAEAG-------      201

                               HR-A-Core    Insert   HR-B
HvHsfB1   ---SASSPTRPDLSSENEQLRKDNHALAAELAMARRHCEELLGFLSRFLDVRQL----DLR------------------------------------------------LLMDEDMQG------AAGGARSG      244
OsHsfB1   ---SASSPPRADITSENEQLRKDNQTLTMELARARRHCEELLGFLSRFLDVRQL----DLR------------------------------------------------LLMQEDMRA------AAGGVGGE      234
HvHsfB2a  ----GAMA---LLEEENARLRRENARLARELARARRVCDGVRHLVWRYDQGGEE----VGVED--------------ER-------------------------------HGAAGGKP-----MLFGVAIGS      222
OsHsfB2a  ---SGAVA---ELEEENARLRRENARLARELARARRVCDGVRRLVSRYDH--D-----HGGGE--------------EE-------------------------------AGEGDVKP-----MLFGVAIGG      219
HvHsfB2b  -SGSGGVASG-DLGEENLRLRRENSRLTRELGQMKKLCNNIFVLMSKYTDGQQV----DAANATSAATDADAGHCSGESAET-MPLP--PPPVLELLPSCPSAPT--AADLGAEDEEEKMSA-RLFGVCIGR      317
OsHsfB2b  -SGSGGVASG-DVGDENERLRRENAQLARELSQMRKLCNNILLLMSKYASTQQL----DAANASSAAGNNNNNNCSGESAEAATPLP--LPAVLDLMPSCPGAAS--AAAPVSDNEEGMMSA-KLFGVSIGR      318
HvHsfB2c  GGAAAGSSSG-DMGEENDRLRRDNARLTRELGQMKKLCNNIVSLMSKFASSQQQ----DGGPGS----LSSVVNCSGES--ALAPPPPLPAAILDLMPSCSALAT---AAGFAVDGGPDADA-RLFGVSIGL      322
OsHsfB2c  GGGGGGSASGGDMGEENERLRRENARLTRELGHMKKLCNNILLLMSKYAATQHV----EGSAG-----ISSIANCSGESSEAVPPPPPLPPAILDLMPSCPALATAAAAAGLAIDGEPDPSA-RLFGVSIGL      327
HvHsfB4b  --ASHGGDFLAALSEDNRQLRRRNSVLLSELAHMKKLYNDIIYFLQNHVAPVTS----PSSAASQR---------------QHPPLPGA----------GASNSCRLLELDMDVDRGSP-------AAEDDD      286
OsHsfB4b  --GGEGGDFLAALSEDNRQLRRRNSLLLSELAHMKKLYNDIIYFLQNHVAPVTTTTTTPSSTAMAA---------------AQHHLP-------------AAASCRLMELDSPDHSPPPPP--PKTPATDGG      278
HvHsfB4c  --GNGRATSAAVLMDENERLRRSNTALLQELSHMRKLYNDIIYFVQNHVRPVAP---SPAAATFLQG---------LGNGMQPRKMPPATTGAGNGLNTSGGSTTSSSSLTIADELSPPPN--HLSAEKSGG      312
OsHsfB4c  --GAGRAATAAVLMEENERLRRSNTALLQELAHMRKLYNDIIYFVQNHVRPVAP---SPAAAAFLQG---------LG--MQARKKP----AAANVLNNSGGSTTSSSSLTIAEEPSPPPQQQHLAGEKSGG      313
```

```
                                  NLS                                                        NES
HvHsfB1   SADQEQCCEKK---------VKLFGVILKD---ASARKRGR------CDEAAAGERSVKMTR---IGEP-------WVGVPSSW-----PAR-------------------------CGGGN       308
OsHsfB1   QRVQEHAREEKC--------VKLFGVLLDDTHGAATRKRAR------CEEAAASERPIKMIR---IGEP-------WVSVPSSG-----PAR-------------------------CGGDN       302
HvHsfB2a KRSREDG--HEG-----------GDEENGAEEDGEDDEEEQE------HNDDDDERHAARRE---QGKSMR-TERSDLNVLSLSVRAAAAAR-------------------------PDGGSRLA    299
OsHsfB2a KRSREENGEDEE-----------EEEEEGADEDGEDDEVEE--------DDEERERHAARRVPVREGKVRRTTELSDLDVLALSVRAAAAAR-------------------------PGGASRDRKSSVS   305
HvHsfB2b KRMRHDGEDQSSR----------GAAEVKP-EPMDAQQPSLDG-----HTTEVQTWPIYRPRP--AYHPLR--ASNGSNCYSGS-----DDH-------------------------NGSSSR  390
OsHsfB2b KRMRHDGGGDDD-----------HAATVKA-EPMDGRPHGKDE-----QSAETQAWPIYRPRP--VYQPIR--ACNGYEYDRAG-----SDQ-------------------------DGSNST  390
HvHsfB2c KRARDEEDEDAVREE--RPNGDGDGADVKPEEAADRRPDGG--------SEERQSWPIYRPKP--VYR-----ACNGQDGAGAG-AGSGSDQ-------------------------DRSNSR  402
OsHsfB2c KRTRDDAAAAADEDGGGEDQAEHGGADVKP-EAADPHPAGGGGGSSTEASPESHPWPIYRPTP--MYHAVR-PTCNGPDRAGSDQDGSSSSQTMGPGEFDDLQKMMVVQQSNFVMHWGRSECGSGVRGFGW  454
HvHsfB4b DT------------------VKLFGVALHH----GKKKRAHG--------------------------------AVAHDLGSEV   316
OsHsfB4b DT------------------VKLFGVSLH-----GRKKRAHRD-----DD-------------------------DGVHDQGSEV   310
HvHsfB4c EAGSSSAALS-AP-------TKLFGVHLSAAPVGAGSKRPPSP-----EEELPSTPPATKAR-----L--V----LESDDLSLSVAPP--------------------------AQPCAASSPARS   388
OsHsfB4c EAGNSSAARSSAP-------TKLFGVHLSAAPCGAGSKRASSP-----EE-HPPTSPATKPR-----L--V----LECDDLSLTVAPSSSSQ---------------------QQLSAASSPTSTS  394
```

C)

```
                               α1              β1        β2     α2                α3                     β3      β4
HvHsfC1a -MDSLHTELALGLIGCGHGD--FQTAPFVAKTYQMVCDPRTDALVRWGKGNNSFLVPDVAGFSQLLLPCFFKHGNFSSFVRQLNTY---------GFRKVHPDRWEFAHESFLRGQTHLLPRIVRRKKRGEAG   121
OsHsfC1a -MDGLHTELALGLIGCCGGDGQQQTAPFVAKTYQMVCDPRTDALVRWGRDNNSFVVVDPAAFSQLLLPCFFKHGNFSSFVRQLNTYVSIIQSPAPGFRKVHPDRWEFAHESFLRGQTHLLPRIVRRKKRGEGG   132
HvHsfC1b -MGSECKGHQHQ--DDGG------VAPFVAKTFHMVSDPATDAVVCWGGASNTFLVLDPAAFSDYLLPSYFKHRNFASFVRQLNTY---------GFRKVDPDMWEFAHESFLRGQAKLLPLIVRKKKRAGAG   115
OsHsfC1b MMGGECKVHQLQAAGDGGPG---AVAPFVAKTFHMVSDPSTNAVVRWGGAGNTFLVLDPAAFSDFLLPSYFKHRNFASFVRQLNTY---------GFRKVDPDRWEFAHESFLRGQAQLLPRIVRKKKKGGAA   121
HvHsfC2a ----MSSAGG------GM------VAPFVAKTYDMVDDPATDSVVAWGPASNSFVVADPFAFSEMLLPAHFKHANFSSFVRQLNTY---------GFRKVDPDRWEFAHSSFLRGQTHLLPRIVRR--RQSGG   106
OsHsfC2a ----MTTTTAEG---GGG------VAPFVAKTYRMVDDPATDGVIAWGRDSNSFVVADPFAFSQTLLPAHFKHSNFSSFVRQLNTY---------GFRKVDPDRWEFAHVSFLRGQTHLLRRIVRR---SSGG   108
HvHsfC2b ----MAAAAASV---GGG------AAPFVWKTYRMVEDPGTDGVIGWGKGNNSFVVADPFVFSQTMLPAHFKHNNFSSFVRQLNTY---------GFRKVDPDRWEFAHGSFLRGQTHLLRNIVRRGTAVAGG  111
OsHsfC2b ----MAAAAG------GG------AAPFVWKTYRMVEDPGTDGVIGWGKGNNSFVVADPFVFSQTLLPAHFKHNNFSSFVRQLNTY---------GFRKVDPDRWEFAHASFLRGQTHLLRNIVRRGSAAAGG  108
```

```
                                                                   HR-A-Core      Insert       HR-B
HvHsfC1a VG---ASCSSAVG-GAEQHQHVVANMGDEVEEEEEEE----------ESEALLEEVQRLRKEQTAIGEQLAKMSRRLQATERRPDRLMSFLARLAEDPSATSLHLFEQAAEKKRQRMQFPSRDFTSLPIALPL   240
OsHsfC1a GGGGGASCSFGGGAGEHQVAAAAASVGMSGEEEDAAED------VLAKEAALFEEVQRLRHEQTAIGEELARMSQRLQATERRPDQLMSFLAKLADDPNAVTGHLLEQAAERKRRRQHLPS--HE--PTVCPL   255
HvHsfC1b AA---------------------G---REVCEEEEEE-----------VRGTIQAVQRLRDERRGMEEELQAMDRRLRAAENRPGQMMAFLGKLADDPGVVLRAMVAKKEE-----------------LAAAG   196
OsHsfC1b PG---------------------C---RELCEEGEE------------VRGTIEAVQRLREEQRGMEEELQAMDQRLRAAESRPGQMMAFLAKLADEPGVVLRAMLAKKEE-----------------LAAAG   201
HvHsfC2a AR----------------------RPSKDDHAEDEDS----------SSAMLAMEVMRLKQEQRATEERVAAMWRRVQDAERRPKLMLAFLLKVVGDP-DVLRRLMGSSSS-----------------DAGLF   189
OsHsfC2a GG----------------------AKRKEEAGGCGGGGEAAAGDVDEESAVVALEVARLRREQREIEGRVAAMWRRVQETERRPKQMLAFLVKVVGDP-QVLRRLVDRDNT-----------------NAAAS   201
HvHsfC2b GG-----------------------KRKDASAADLTG---------DDMTMVATEVVRLKKEQSTIDDRVAAMWRRVQETERKPKQMLAFLLTIVGDR-DTLQRLVANSGN-----------------AASGD   194
OsHsfC2b GGGG------------------GGGKRRDASADGGGGG------GDEDMTMVATEVVRLKQEQRTIDDRVAAMWRRVQETERRPKQMLAFLLKVVGDR-DKLHRLVGGGGNGNGAA-----------TAAAAD  205
```

```
HvHsfC1a Q---PAPSP--PPP-PLLALG----------------------------DAAMGGVRVW-QWAEP----IPLK-----LTTFEQPSASS----GVQQVPEFE                                  294
OsHsfC1a P---PAPPP--QPPQPLLALAG---------------------------AAAMDGTYWW-TTEHHHHHHHQMKPMT-VLPSLEPPTASC----GVHQVPELGGGGVMGLTTDGEAKVEPPFPFCLLGQAFF   348
HvHsfC1b A---GGMDP--RPD-KRRRIGA---------------------------------------EAEEA---AQSR---------AVPFPFS----NAGHVFY                                   235
OsHsfC1b N---NGSD----PC-KRRRIGA---------------------------DTGRGGVATG-GDAAEM---AQSRG--------TVPFPFS----VLGQVFY                                   250
HvHsfC2a P--GDGAEP----KRPRLLLDG----EVHVGKKMRVDG------DGMLYGTDRQDVFVREPGADFAGFYTGGDGFS--DVPVDDPP----------YVFPVDSGY                                266
OsHsfC2a N--ADDSAVHHQVKRPRLLLDSSSTTTTHGDRHLVTAAADGFYAGGCGPEAAAAAAFVPDDAVDFTGLYTGGDGFG--NAVVDAGVDYP---PAYAFPV-VDSGY                               298
HvHsfC2b Q---EPVEG--GEKRARLLLDG---------------------------DFGNVSAFGP-DAVDFAGFYADGDAFANVPVPVEAAAGSGGGGAGCSFAFGVDSGY                                266
OsHsfC2b NGFADAARAGCGEKRARLLLDG---------------------------D--NTGAFGP-DAVDFAGFYTGADMFP--DVAVDAAAAAAGGSAGCSFAFGVDSGY                               278
```
