# Supplementary material for: Unraveling Regulation of the Small Heat Shock Proteins by the Heat Shock Factor HvHsfB2c in Barley: Its Implications in Drought Stress Response and Seed Development
Source: PLoS One. 2014 Mar 4;9(3):e89125. doi: 10.1371/journal.pone.0089125 (PMC3942355; doi:10.1371/journal.pone.0089125)
Supplement: Figure S2 — Multiple alignment of different sub-classes of Hv sHsps. The conserved α- crystallin domain was labeled with dotted box. The defined consensus regions I and II are marked with underline below the sequences. Highly conserved and semi-conserved regions are shown in “*” and “.”, respectively. Small Hsp region specific to respective subclasses was labeled with thick boxes. The chloroplast localized proteins have transit sequences that are specific for organelle and is labeled with red colour. The conserved Arg is displayed in red colour in the β7 strand. The secondary structure assignments for all classes of HvsHsps were labeled above the sequences. The predicted β-strands depicted by thick lines above the alignment are based on their position in known secondary structure of Hsp16.9 from T. aestivum (van Montfort et al. 2001). The IXI/V motif in the C-terminal extension is shown in green. The SXXFD motif and interacting residues in the conserved alpha crystallin domain are in pink colour. Alignment was performed by using ClustalW (DNAstar) program. (PDF) [file pone.0089125.s002.pdf]

Figure S2

a) CI

```
                                                                  β2      β3           β4        β5               β6        β7            β8       β9                   β10

WHsp16.9-CI      MSIVRRTNVFDPFA-DLWA--D--PFD------TFRSIVP---AISGGGSETAAFANARMDWKETPEAHVFKADLPGVKKEEVKVEVEDGNVLVVSGERTKEKEDKNDKWHRVERSSGKFVRRFRLLEDAKVEEVKAGLENGVLTVTVPKAEVKKPEVKAIQISG   151
HvHsp16.9-CI     MSIVRRSNVFDPFA-DLWA--D--PFD------TFRSIVP---AISGGNSETAAFANARMDWKETPEAHVFKADLPGVKKEEVKVEVEDGNVLVVSGERTKEKEDKNDKWHRMERSSGKFVRRFRLPEDAKVEEVKAGLENGVLTVTVPKAEVKKPEVKAIEISG   151
HvHsp16.86-CI    MSIVRRSNVLDPFA-DLWA--D--PLD------TFRSIFP---AISGGNSETAAFANARMDWKETPEAHVFKADLPGVKKEEVKVEVEDGNVLIVSGERTKEKEDKNDKWHRVERSSGKFVRRFRLPEDAKVDEVKAGLENGVLTVTVPKAEVKKPEVKAIEISG   151
HvHsp16.88-CI    MSIVRRSNAFDPFA-DLWA--D--PFD------TFRSIVP---AFSG-NSETAAFANARVDWKETPEAHVFKADLPGVKKEEVKVEVEDGNVLVVSGERTKEKEDKNDKWHRVERSSGKFVRRFRLPEDAKVEEVKAGLENGVLTVTVPKTEVKKPEVKAIEISG   150
OsHsp169A-CI     MSLVRRSNVFDPFSLDLW---D--PFDS-----VFRSVVP---ATSD--NDTAAFANARIDWKETPESHVFKADLPGVKKEEVKVEVEEGNVLVISGQRSKEKEDKNDKWHRVERSSGQFMRRFRLPENAKVDQVKAGLENGVLTVTVPKAEVKKPEVKAIEISG   150
OsHsp169B-CI     MSLVRRSNVFDPFSLDLW---D--PFDS-----VFRSVVP---ATSD--NDTAAFANARIDWKETPESHVFKADLPGVKKEEVKVEVEEGNVLVISGQRSKEKEDKNDKWHRVERSSGQFMRRFRLPENAKVDQVKAGMENGVLTVTVPKAEVKKPEVKAIEISG   150
OsHsp169C-CI     MSLVRRSNVFDPFA-DFW---D--PFDG-----VFRSLVP---ATSD--RDTAAFANARVDWKETPESHVFKADLPGVKKEEVKVEVEEGNVLVISGQRSKEKEDKNDKWHRVERSSGQFMRRFRLPENAKVDQVKASMENGVLTVTVPKAEVKKPEVKAIEISG   149
HvHsp16.7-CI     MSLIRRGDVFDPFSLDLWD-----PFGSG-----SGSVFP---RTTD--SETAAFAGARIDWKETPDAHVFKADVPGLRKEEVKVEVDDGNVLQISGERNKEQEEKTDTWHHVERSSGRFLRRFRLPDNARAEEIKAAMENGVLTVTVPKAEAKKPDVKPVQITG   150
HvHsp17.7-CI     MSLIPRGNAFDPFSVDLWNPFDGFPFGSG-SSSSGGSLFPSFPR-TS--SDTAAFAGARIDWKETPEAHVFKADVPGLKKEEVKVEVEDGNILQISGERNKEQEEKTDTWHRVERSSGKFLRRFRLPEDAKADQIKAAMENGVLTVTVPKEEAKKPEIKSIQISG   161
HvHsp17.5-CI     MSLIRRSNVFDPFSLDFFDPFDGFPFGSG-SSNSGGSLVP---R-TS--SDTAAFAGARIDWKETPEAHVFKADVPGLKKEEVKVEVEDGNILQISGERNKEQEEKTDTWHRVERSSGKFLRRFRLPENAKAEQVKASMENGVLTVTVPKEEAKKPEVKSIQISG   158
OsHsp174-CI      MSMIRRSNVFDPFSLDLWDPFDGFPFGSG-----SGSLFP---R-AN--SDAAAFAGARIDWKETPEAHVFKADVPGLKKEEVKVEVEDGNVLQISGERIKEQEEKTDKWHRVERSSGKFLRRFRLPENTKPEQIKASMENGVLTVTVPKEEPKKPDVKSIQITG   154
OsHsp179A-CI     MSLIRRSNVFDPFSLDLWDPFDGFPFGSG--GSSSGSIFPSFPRGAS--SETAAFAGARIDWKETPEAHVFKADVPGLKKEEVKVEVDDGNILQISGERNKEQEEKTDQWHRVERSSGKFLRRFRLPDNAKPEQIKASMENGVLTVTVPKEEAKKPDVKSIQISG   161
OsHsp180-CI      MSLIRRSNVFDPFSLDLWDPFDGFPFGSG--SRSSGSIFPSFPRGTS--SETAAFAGARIDWKETPEAHVFKADVPGLKKEEVKVEVEDGNVLQISGERSKEQEEKTDKWHRVERSSGKFLRRFRLPENTKPEQIKASMENGVLTVTVPKEEPKKPDVKSIQVTG   161
OsHsp177-CI      MSLIRRGNAFDPFSLDLWDPVDGFPFGSGGSSSSSGSLFP---R-AN--SDAAAFAGARIDWKETPEVHVFKADVPGLKKEEVKVEVDDGNILQISGERSREQEEKSDKWHRVERSSGKFLRRFRLPENTKPEQIKASMENGVLTVTVPKEEPKKPDVKSIQISG   159
                 **...*....***.**.*.*.****..****.....*..****....*....****.**.******..******.**..********..**.*..**.*..*.*.*.*.***.*******.*****.*.......**..***********.*.***........*
                                                                                          Consensus Region II                    Consensus Region I
```

b) CII

```
                                                                  β2         β3           β4            β5               β6        β7              β8     β9

HvHsp17.7-CII    MEGRMFGLETPLMTALQHLLD--IPDGEAGGVGVAG--GEKQGPTRAYVRDARAMAATPADVKEL--PGAYAFVVDMPGLGSGDIKVQVEDERVLVISGERRR-----EEKED-AKYVRMERRMGKMMRKFVLPENADMEKIAAACRDGVLTVTVEKLPPPEPKKP   154
HvHsp17.77-CII   MEGRMFGLETPLMTALQHLLD--IPDGEAGGPGHAG--GEKQGPTRAYVRDARAMAATPADVKEL--PGAYLFVVDMPGLGSGDIKVQVEDERVLVISGERRR-----EEKED-ARYLRMERRMGKLMRKFVVPDNADTEKISAVCRDGVLTVTVDKLPPPEPKKP   154
HvHsp17.3-CII    MAGMVFGLETPVMTALQHLLD--IPDGEAG---AAG--GEKQGPTRAYVRDARAMAATPADVKEL--PGAYAFVVDMPGLGSGDINVQVEDERVLVISGERRR-----EEKED-AKYVRMERRMGKMMRKFVLPENADMEKISAACRNGVLTVTVEKLPPPEPKKP   151
OsHsp180-CII     MESAMFGLETPLMTALQHLLD--IPDGEGG---AAGKQGATGGPTRAYVRDARAMAATPADVKDL--PGAYAFVVDMPGLKSSDIKVQVEEERLLVISGERRRGGGE-EEKEESCKYLRMERRMGKFMRKFVLPDNADVDKISAVCQDGVLTVTVEKLPPPEPKKP   158
OsHsp190-CII     MELSVVGGEPLLAVAMQQLLDLDLPDELER---------QLNPPTRAYVRDRRAMANTPMDVKELRASGALVLAVDMPGVAPADVRVEVEDGNVLAISGERRRPAGDGDDGGEGVKYLRMERRMGKFMRRFPLPESADLDGVRAEYKDGVLTVTVDKKPPPEPKKP   157
                 *.....*.*.....*.*.********....*.*.*****....************.**.***.***.**....*****....*..*.**....*.*******..*..........*.********.****..*..**.....*....*******.*.********
                                                                                    Consensus Region II                    Consensus Region I

                    β10

HvHsp17.7-CII    KTIQVQVA                                                                                                                                                              162
HvHsp17.77-CII   KTIQVQVA                                                                                                                                                              162
HvHsp17.3-CII    KTIQVQVA                                                                                                                                                              159
OsHsp180-CII     KTIEVKVA                                                                                                                                                              166
OsHsp190-CII     RVVEVKVAGAGEPKGKGK                                                                                                                                                    175
                 ......************
```

c) CIII

```
                                                                    β2      β3           β4      β5              β6         β7         β8             β9       β10

HvHsp190-CIII    MAELLFAPAMAGLVHLPEVLDRLAAADADHR---DHAHHHAAHGHGHAHPRAHIGAGGGAPVDIVETPGEYSFLLDVPGLSKSDIQVTLEEDNVLVMKSASSSNGGGANGKRKREEEEADCRYIRLERRASPRSFVRKFRLPEDADAGAVAARCENGVLTVTVKKQ   163
OsHsp186-CIII    MTEL-FDTAVTSLLHLPEVLDRLGAAAGDRRSAGDHAHH-AAHGHGQHR---ISGIGGGAPVDIMETPGEYAFVLDVPGLSKSDIQVTLEEDRVLVMK---SSNGAG-NGKRKREEEEGECKYIRLERRASPRAFARKFRLPEDADTGGISARCENGVLTVTVKKR   157
                 *.****..*...*.*********.**....*********.******........*.*********.******.*.******************.*****...****.*.**********..*.***********.*.**********.*...*************.
                                                                                            Consensus Region II                               Consensus Region I

                          β11

HvHsp19.0-CIII   PPPEKKTKSVQVAIA                                                                                                                                                       178
OsHsp186-CIII    PPPEKKTKSVQVTIA
                 ************.**                                                                                                                                                       172
```

d) CV

```
                                                                                β2      β3                β4          β5              β6        β7               β8        β9
HvHsp17.1CV MD--YPMQ---EVQQHPGFR-AVHPWQWWQWQ--LSVLASSSSPPAPSTRPP--NHVSWDETGAAHIYSADLPGVKKEEIRVEIEDGRYLVVRTELDAG---DAEVHGRRGGFARKFRLPGMVDADGITAEYAHGVLTVTVPRMHNRARPMVNLIGSG-----PAC      148
OsHsp188-CV MDYYYPMEEEEEVHERPRFRRPVHPWQWHHWQNLLGLLSSSSPSPATAAAAQRCSHVSWEETAAAHLYSASLPGVRKEEIRVEVEDAMYLVIRTELDDGGDGDGGGGGGRRSFARKFRLPAMVDADGISAEYTHGVLRVTVPRLHTRARPVVNLAGGGGGGGGPAC      166
            **..***....**...*.**..******..**..*..*.***..**.........****.**.*******************.**..***.*****.*...*....*.*..********.*******.***.****.*****.*.****.***.*.*.....***

                                                                                         Consensus Region II                                   Consensus Region I

HvHsp17.1CV DPVARAA                                                                                                                                                                155
OsHsp188-CV DPVARAA                                                                                                                                                                173
            *******
```

e) CIX

```
                                                                            β2      β3                β4          β5              β6        β7               β8        β9                        β10
HvHsp17.76-CIX MSMVRFLDTLALDSWNPFGSIFGTTASGGADAWLASDTTAFADTYIESRETAEAYVFSARLPAGVTKEEVKVEVEEEGKVLVIAGERTLRREAKNETRHHVERSCATFFGRFHLPEDAALGRVRAAMDDGGAQLTVTVPRVG--------AAAAAEAMPEPAVAIE      158
OsHsp179B-CIX  MSLVKLFDTLAFDAWNPF-SIFGTTVA--ADAWLASDTSAFANTYIESRETAEAYVFRADLPAGVKKEEVRVEVDE-GNVLVITGERSVRREEKGQRSHHIERSCATFFGRFHLPDDAVVDLVRASMDGG--MLTVTVPKVVTDKQPAIAAAAPVPAVVAPAVEAK      160
               **.*...****.*.****.******....*********.***.**************.*.*****.****.***.*.*.****.***..***.....**.****************....******.....******.*.........***...*...***...
                                                                                 Consensus Region II                                   Consensus Region I

HvHsp17.76-CIX VVEASPC                                                                                                                                                             165
OsHsp179B-CIX  AIEASP                                                                                                                                                              166
               ..****.
```

f) CX

```
                                                                                                β2      β3                β4          β5              β6        β7               β8        β9
HvHsp19.2-CX MSMISNMLGRRQPPPPQQQQPAGHKTNGHGGGGEVVEPVSIDILEPFMEAISLKAFGGP------ALGLPFSTASMDWKETPTAHVFMADVPGLRREEVKVEVEQERVLRISGQRARAAEDKGDRWHRVERSAEKFVRTVRLPPNADVDGGGVHAALDNGVLTITI      160
OsHsp188-CX  MSMITSMLGRKQN--AQQKGGGGGGRTGGGGGGE-IEPVSVDIMEPFMDAISLTAFAAAPSAAAAAAGVP-STASMDWKETAAAHVFMADMPGVRREEVRVEVEEEKVLRISGQRARAAEEKGERWHRVERSSERFVRTVRLPPNANTDG--VHAALDNGVLTITI      160
             ****..******....**.........*.*****..****.**.****.****.**.........*.*.*.**********..**********.*****.****.*.*************.**.********.*.***********..**..*************
                                                                                                                   Consensus Region II                                   Consensus Region I

                  β10
HvHsp19.2-CX PKDDGKKAYGRIIPITN                                                                                                                                                      177
OsHsp188-CX  PKDNDRKPHARIIPITN                                                                                                                                                      177
             ***...*...*******
```

g) Px

```
                                                           β2      β3                β4          β5              β6        β7               β8        β9                β10
HvHsp15.1-Px MADLFFGGSPFRRLLYARPL------ASAPGAMDWVETQTSHVLRINVPGLGKDDVKVQVEDGNVLSVRGAAKEKTKEGN---EEDAVWHVSERGKPEFAREVPLPEHVRVDQIRASVDNGVLTVVVPKEPAPARPRTRPITVSSKL      138
OsHsp160-Px  MADLFFGG-PFRRILYGRPFPPDWASASATAAMDWVETPTSHVLRINVPGLGKDDVKVQVEDGNVLTVRGAAPHAAAEKEREREKDVVWHVAERGRPEFAREVALPAEVRVEQIRASVDNGVLTVVVPKEPAPARPRTRPIAVSSKL      146
             ********.*******.**.......***..*******.*************************.*****.....*.....***.****.***.*******.**..***.***************************.*****
                                                Consensus Region II                                      Consensus Region I
```

h) M

```
                                                                                                                                                                                   β2       β3
HvHsp21.3-MI  --MASAVACKGATPASFLK-----------SGAPVAFCPLN-----------TTAVTADHRPYNTLVKEAIRYD--DD------DDYS----GRHLVLPSFFSQ-DVLDPLG------------------AHQTGLS--CTAGASRLGR-WVTKEDDGAVYLKVP   107
OsHsp24.0-MI  --MASIVASKRIPLFRLVEQLLAA------SPAQGAASALR-----------PVAVAGGSRAYNTG-AQLRRHER-DESDDDSGRGYDTRRPTRDATMPAFFSGNVFRDPFSAPQSLGRLLSLMDD----LATPAGRAG---AATLRRG--WNAKESEEALHLRVD   136
OsHsp26.2-MI  --MASTVALKGRPLATLLRQLLAADAPPAATGRPVAAAPAASGKPVTAPAAATATNAASRRLYNTEGAPLRRYDVVDESGTDSGDEYDATDDGRRLTVPFFFSASDVLDPFGAPTSLGRLLALMEDAAVATAAAPGTNGLATAAARRGGW-WVAKEDDDAVHLKVS   163
OsHsp23.6-MII MALARQCLSKRLAAGCALARPLH-------AASPVAAAAAN-----------SHGPLNFRALFSSAGADAAATT--GGCAPAKGDGHSREVAVVDRSRRRWPWR-DLRDFVP-----------LR-----LVDGIGSALSQVAETLTRPLTGKVREDEERYRLRFE   129
              ...*.............................*......................................................*......******..............*......*...........*......*...

                        β4         β5                          β6              β7         β8             β9
HvHsp21.3-MI  MPGLTKEHVQVRADKNILVIKGE------GEKQPWAGDDDSAVPRYNHRIEIPADAYKMDKIKAEMKNGMLWVTLLKLKEEERKDVFHVKVE   193
OsHsp24.0-MI  MPGLGKEHVKVWAEQNSLVIKGE------GEKEA-GEDEGAAPARYSGRIELAPEVYRMDQIKAEMKNGVLKVVVPKVKEEQRRDVFQVNVE   221
OsHsp26.2-MI  MPGLGKEHVKVWAEQNSLVIKGE------GEKDP-EDDADAAPPRYTRRIELPADAFKMDKIKAEMKNGVLRVAVPKLKEEERKDVFQVNVE   248
OsHsp23.6-MII VPGLGKDDVRVYVDDGVLAIHGEKRDVVEEDRGRDGDGECWAAATYHAGLLLPEDAVAEG-ITAEVRDGVLHVTVPRS-PERKRSVTEVKVR   219
              .***.*..*.*......*.*.**.....................*.....*.........*.**...*.*.*.....*.*.........*.
                Consensus Region II                                     Consensus Region I
```

i) ER

```
                                                                                                                                            β2         β3             β4             β5                   β6         β7
HvHsp-ER      ----------------------------------------------------------------------------------------------------------------NDQPHF--------------------------------------WTK-ERSIGDFQRTFSFTARVD    27
HvHsp21.9-ER  MAAVSKK---ALVATAVLALAVLAPPVAGLVPYGVSSG--------LWDLLDDPFRVLEQAPLAVQRPASAGDPAASVSSPMALARCDWKETPDAHVISLDVPGVRRDDVKEVEEN-RVLRVSGERK---ADEEKEGER----WHRAERAAGRFWRRFRMPAGAD   147
OsHsp232-ER   MASMRTAAAAAMLACIAVVLASTAADGALLPWFGGGGARDEAVPELGLLAAADPFRILEHVPFGFDR---------DDVAMLSMARVDWRETGDAHEVVVDVPGMRKEDLRVEVEDN-RVLRISGERRREETTEQKGGGD---HWHREERSYGRFWRQLRLPDNAD   153
OsHsp218-ER   MAAVAEREVLGMVAAVAAMVVMMAPPAAALVPYGYG-----------YMLDDPFRVLEQSPLRPAG-GVAAAAAAGEPAAVALARCDWKETPEAHVVTVDVPGVRRGDVRVEVDEASRVLRVSGERRRAGAAEEEEGERDGVRWHRAERAAGRFWRRFRMPPGAD   153
              **..........*.........*...*.*...*.................****.**..*......................**.**.**..**....*.*..*..*..***....****.****.*....*...*......*...**..*.*.*........*
                                                                                                                                   Consensus Region II

                 β8       β9              β10
HvHsp-ER      QDSVRASLKNGVLSVVVPKEAAPTPKKIR                                          56
HvHsp21.9-ER  VERVTARLEDGVLTVTVPKIAEHQRREPRVINIAGEASNNGGK----GAGAGAEVTSSKSEMM       206
OsHsp232-ER   LDSIAASLDNGVLTVRFRKLAPDQIKGPRVVGIASAGGDDGGKKSIGGAGEGQNQQAKKVEL       215
OsHsp218-ER   VGRVAARLDDGVLTVTVPKVPGHRGREPRVVAIDG-----AGA----GDMEAEVVKASKAEM       206
              .....*.*..***.*...*..........**..*.........*.....*...........*.*..
              Consensus Region I
```

j) P

```
                                                                                        β2                                                                                        β3       β4
HvHsp26.8-P MAAATAPFALVSRLSPAARLPIR-AWRAARPAP--LWTGRTRPLSVASAAQENTD-SSVDVQVSQDRNAGNQQGNAVQRRPRRAAGFD-ISPFGLVDPMSPMRTMKQMLDTMDRLFDD-AVGFPTAGRSPAAAAGEMPRMPWDIMEDDKEVKMRFDMPGLSREEVK   160
OsHsp26.7-P ---MAAPFALVSRVSPAARLPIRAAWRRARPTVGLPSSGRARQLAVASAAQENRDNTAVDVHVNQD--GGNQQGNAVQRRPRRSSALDGISPFGLVDPMSPMRTMRQMLDTMDRIFDDVALGFPATPRR-SLATGEV-RMPWDVMEDDKEVRMRFDMPGLSREEVK   159
            .....********.*********.*******.......**.*.*.********.*...***.*.**...**************......****************.************.*.***...*....*.**..*****.**********************

             β5      β6                          β7                    β8        β9           β10
HvHsp26.8-P VMVEDDALVIRGEHKKEAGEGQGEAAGGGDGWWKERSVSSYDMRLALPDECDKSQVRAELKNGVLLVSVPKRETERKVIDVQVQ   244
OsHsp267-P  VMVEDDALVIRGEHKKEEGEG---AEGSGDGWWKERSVSSYDMRLALPDECDKSKVRAELKNGVLLVTVPKTEVERKVIDVQVQ   240
            *****************.***...*.*.**************************.************.***.*.**********
            Consensus Region II                                   Consensus Region I
```
