# Supplementary figures and images for: Unraveling Regulation of the Small Heat Shock Proteins by the Heat Shock Factor HvHsfB2c in Barley: Its Implications in Drought Stress Response and Seed Development
Source: PLoS One. 2014 Mar 4;9(3):e89125. doi: 10.1371/journal.pone.0089125 (PMC3942355; doi:10.1371/journal.pone.0089125)

Figure S3

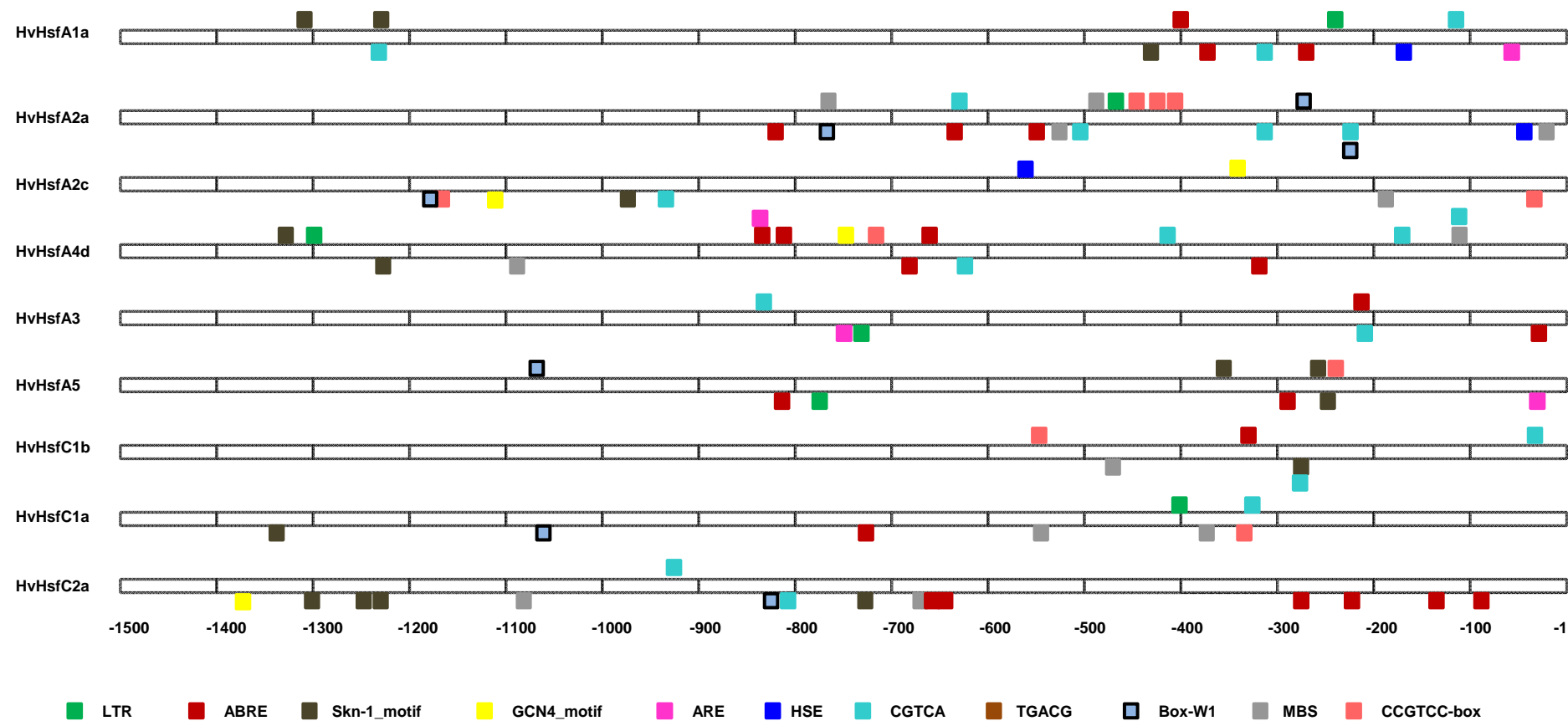

Supplement: Figure S3 — Position of putative cis -elements present in the promoter regions of barley Hsf genes. The analysis was performed using PlantCARE and PLACE databases. The “rectangle mark” shows the relative position of the different motifs. (PDF) [file pone.0089125.s003.pdf]

Figure S4

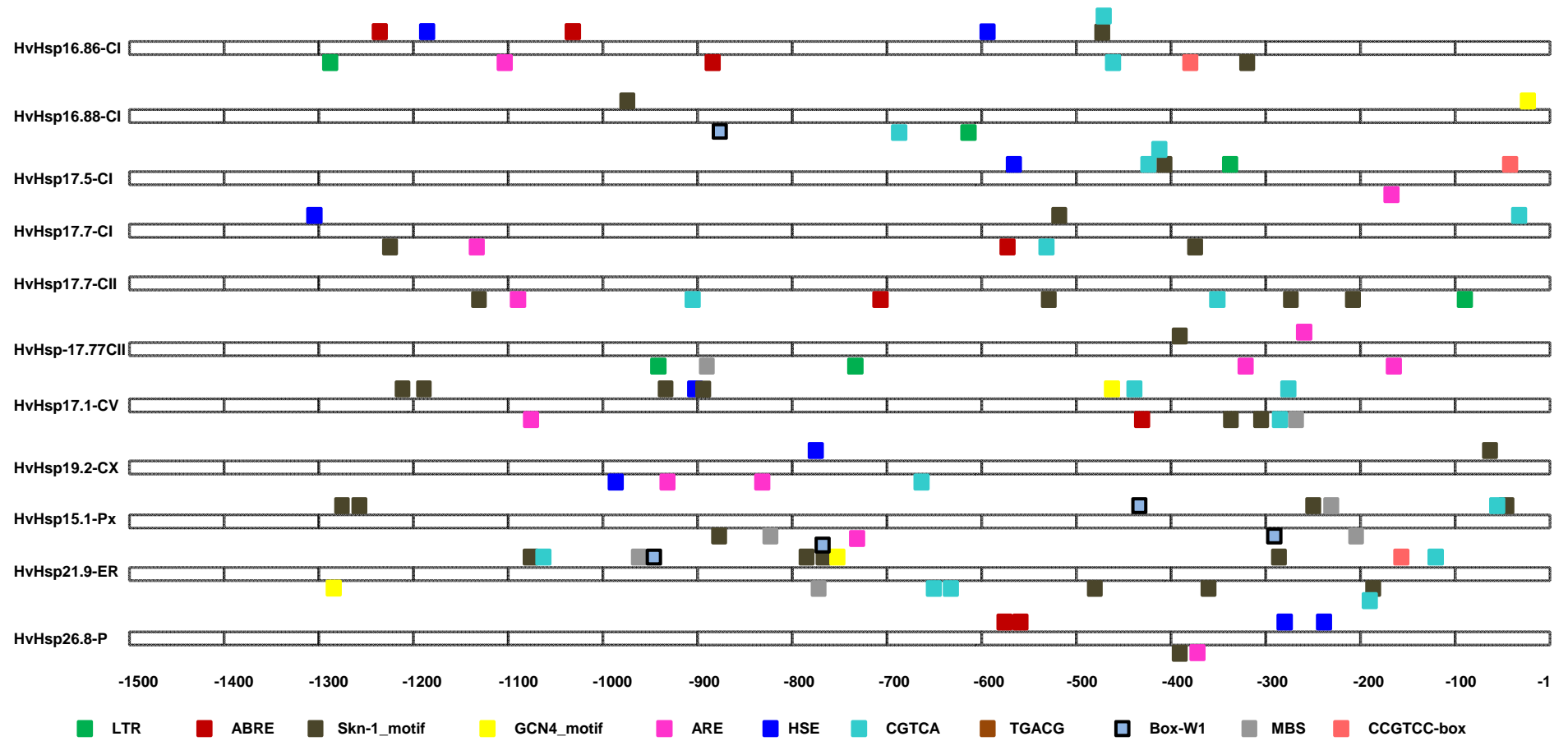

Supplement: Figure S4 — Position of putative cis -elements present in the promoter regions of barley sHsp genes. The analysis was performed using PlantCARE and PLACE databases. The “rectangle mark” shows the relative position of the different motifs. (PDF) [file pone.0089125.s004.pdf]
