# Supplementary material for: Unraveling Regulation of the Small Heat Shock Proteins by the Heat Shock Factor HvHsfB2c in Barley: Its Implications in Drought Stress Response and Seed Development
Source: PLoS One. 2014 Mar 4;9(3):e89125. doi: 10.1371/journal.pone.0089125 (PMC3942355; doi:10.1371/journal.pone.0089125)
Supplement: Figure S5 — Subcellular localisation of HvHsfB2c in transient transformed Arabidopsis thaliana mesophyll protoplasts for the the N-terminal CFP-fusion derivatives. scale = 10 µM. A, N-terminal CFP-fusion; B, C-terminal CFP fusion. (PDF) [file pone.0089125.s005.pdf]

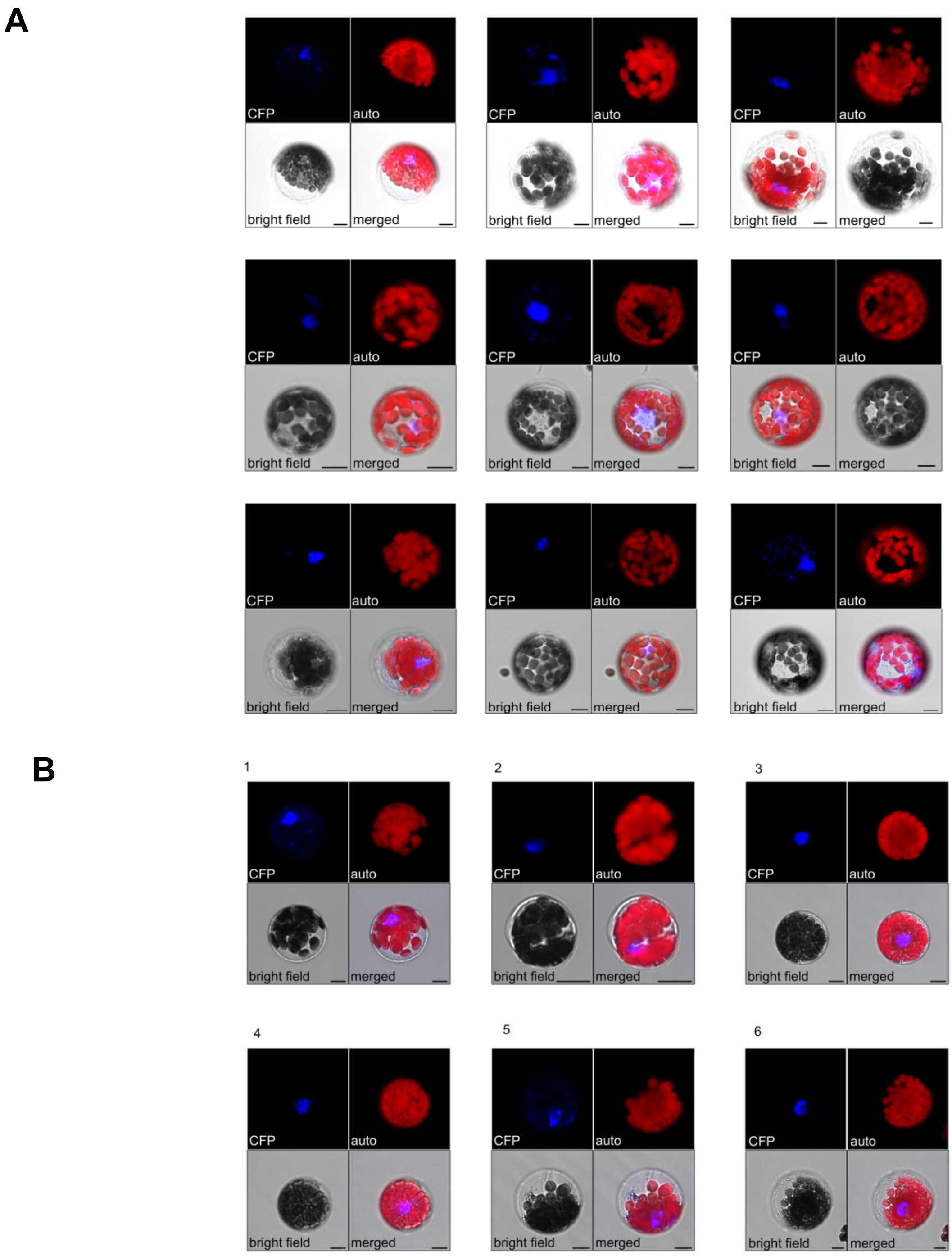

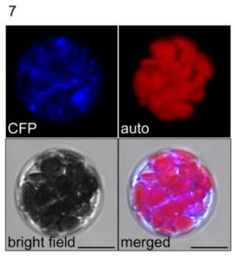

Figure S5.
Subcellular localisation of HvHsfB2c in transient transformed *Arabidopsis thaliana* mesophyll protoplasts, scale = 10 µM. A, N-terminal CFP-fusion; B, C-terminal CFP fusion.
