# Supplementary material for: Unraveling Regulation of the Small Heat Shock Proteins by the Heat Shock Factor HvHsfB2c in Barley: Its Implications in Drought Stress Response and Seed Development
Source: PLoS One. 2014 Mar 4;9(3):e89125. doi: 10.1371/journal.pone.0089125 (PMC3942355; doi:10.1371/journal.pone.0089125)
Supplement: Table S1 — Barley sHsp and Hsf Orthologous genes in Arabidopsis . (PDF) [file pone.0089125.s006.pdf]

Table S1. **Barley sHsp and Hsf Orthologus genes in Arabidopsis**

S.No	Query sequence	Subject sequence	Description of subject	Identity
1	HvHsp16.9-CI	At5g59720	HSP18.2 (Heat shock protein 18.2)	87/150 (58%)
2	HvHsp16.88-CI	At5g59720	HSP18.2 (Heat shock protein 18.2)	89/150 (59%)
3	HvHsp26.8-P	At4g27670	HSP21 (heat shock protein 21)	99/198 (50%
4	HvHsp19.0-CIII	At1g54050	Unknown protein	56/120 (46%)
5	HvHsp17.76-CIX	At5g59720	HSP18.2 (Heat shock protein 18.2)	45/123 (36%)
6	HvHsp17.1CV	At3g46230	AtHSP17.4	43/86 (50%)
7	HvHsp19.2-CX	At3g46230	AtHSP17.4	62/155 (40%)
8	HvHsp16.7-CI	At5g59720	HSP18.2 (Heat shock protein 18.2)	91/149 (61%)
9	HvHsp21.3-MI	At4g25200	AtHsp23.6-mito (Mitochondrion-localized small heat shock Protein 23.6)	77/220 (35%)
10	HvHsp17.5-CI	At3g46230	ATHSP17.4	77/117 (65%)
11	HvHsp17.7-CI	At3g46230	ATHSP17.4	93/167 (55%)
12	HvHsp17.7-CII	At5g12030	AT-HSP17.6A	71/154 (46%)
13	HvHsp17.77-CII	At5g12030	AT-HSP17.6A	79/154 (51%)
14	HvHsp21.2	At1g54400	unknown protein	32/68 (47%)
15	HvHsp17.3-CII	At5g12030	AT-HSP17.6A	71/151 (47%)
16	HvHsp-ER	At5g12030	AT-HSP17.6A	20/54 (37%)
17	HvHsp	No significant hit were obtained		
18	HvHsp15.1-Px	At5g37670	unknown protein	66/143 (46%)
19	HvHsp16.86-CI	At5g59720	HSP18.2 (Heat shock protein 18.2)	85/150 (56%)
20	HvHsp21.9-ER	At4g10250	ATHSP22.0	65/148 (43%)
21	HvHsfA5	At4g13980 _2	probable heat shock transcription factor - Arabidopsis thaliana.	184/454 (40%)
22	HvHsfB2b	At4g11660	AT-HSFB2B; transcription factor	141/380 (37%)
23	HvHsfA4d	At4g18880	AT-HSFA4A; DNA binding / transcription factor	160/440 (36%)
24	HvHsfA4b	At4g18880	AT-HSFA4A; DNA binding / transcription factor	165/442 (37%)
25	HvHsfC1b	At3g24520	AT-HSFC1; DNA binding / transcription factor	85/182 (46%)
26	HvHsfB2c	At4g11660	AT-HSFB2B; transcription factor	85/101 (84%)
27	HvHsfA2d	At3g22830	AT-HSFA6B; DNA binding / transcription factor	98/266 (36%)
28	HvHsfC2b	At3g24520	AT-HSFC1; DNA binding / transcription factor	94/193 (48%)
29	HvHsfA1a	At3g02990	ATHSFA1E; DNA binding / transcription factor	211/496 (42%)
30	HvHsfA2a	At2g26150	ATHSFA2; DNA binding / transcription factor	126/218 (57%)
31	HvHsfA9	At1g32330	heat shock transcription factor family [Arabidopsis thaliana].	108/244 (44%)
32	HvHsfB4c	At1g46264	AT-HSFB4; DNA binding / transcription factor	132/252 (52%)
33	HvHsfA2c	At3g22830	AT-HSFA6B; DNA binding / transcription factor	172/359 (47%)

34	HvHsfA3	At5g03720	heat shock transcription factor -like protein [Arabidopsis	115/219 (52%)
35	HvHsfA2b	At3g22830	AT-HSFA6B; DNA binding / transcription factor	153/251 (60%)
36	HvHsfA2e	At3g22830	AT-HSFA6B; DNA binding / transcription factor	164/325 (50%)
37	HvHsfB2a	At4g11660	AT-HSFB2B; transcription factor	67/92 (72%)
38	HvHsfC1a	At3g24520	AT-HSFC1; DNA binding / transcription factor	89/202 (44%)
39	HvHsfC2a	At3g24520	AT-HSFC1; DNA binding / transcription factor	94/172 (54%)
40	HvHsfA2f	At2g26150	ATHSFA2; DNA binding / transcription factor	92/175 (52%)
41	HvHsfB4b	At1g46264	AT-HSFB4; DNA binding / transcription factor	145/296 (48%)
42	HvHsfB1	At4g36990	HSF4 (Heat shock factor 4); DNA binding / transcription factor	98/269 (36%)
