# Supplementary material for: Unraveling Regulation of the Small Heat Shock Proteins by the Heat Shock Factor HvHsfB2c in Barley: Its Implications in Drought Stress Response and Seed Development
Source: PLoS One. 2014 Mar 4;9(3):e89125. doi: 10.1371/journal.pone.0089125 (PMC3942355; doi:10.1371/journal.pone.0089125)
Supplement: Table S2 — Additional file 5: Detailed list of functional annotations of genes represented in the network of HvHsfB2c/HvHsp17.5-CI . (PDF) [file pone.0089125.s007.pdf]

ProbesetID	TranscriptID		Annotation	MapManTern	Consensus E
HVSMEi0006K11r2_at	bab91939.1		(ap003768) putative nadp dependent malic enzyme [oryza sativa (japonica cultivar_group)]	8.2.10	35_961
Contig6860_at	bab08888.1		gene_id:mij24.6~ref np_013897.1~similar to unknown protein [arabidopsis thaliana]	35,2	35_16191
Contig18997_at	aam22740.1		(ac092388) putative serine/threonine protein kinase [oryza sativa (japonica cultivar_group)]	29.4.1.57	35_7561
rbags10k08_at	bac15853.1		(ap003746) contains ests d25106(r3184) au184634(r3184)~diphosphonucleotide	35,2	35_8534
HY06D12u_s_at	hy06d12u_s_at		no annotation	35,2	35_17271
Contig21187_at	np_197879.1		(nm_122406) putative protein; protein id: at5g24970.1 [arabidopsis thaliana]	35.1.1	35_24327
HVSMEa0007I03r2_at	baa95828.1		ests d47168(s12332) d46350(s10967) correspond to a region of the predicted gene.~similar to prunus armeniaca	17.5.1	35_43599
rbah15i08_s_at	np_195066.1		dem _like protein; protein id: at4g33400.1 supported by cdna: gi_20465422 [arabidopsis thaliana]	33,99	35_18661
HV_CEb0006P16r2_s_at	hv_ceb0006p16r2_s_at		no annotation	35,2	35_4008
Contig8246_at	aak27799.1		(ac022457) putative amylase [oryza sativa (japonica cultivar_group)]	2.2.2.1	35_17258
Contig21613_at	bab89059.1		(ap003290) pdi_like protein [oryza sativa (japonica cultivar_group)]	35,2	35_25874
Contig15315_at	aag41776.1		(af212990) ent_kaurene oxidase [cucurbita maxima]	17.6.1.3	35_21965
Contig10520_s_at	np_176898.1		integral membrane protein putative; protein id: at1g67300.1 [arabidopsis thaliana]	34,2	35_4761
Contig7750_at	np_200309.1		putative protein; protein id: at5g54980.1 [arabidopsis thaliana] dbj bab08777.1 gb aad50013.1~gene_id:mbg8.25~similar	35,2	35_17906
Contig9045_at	bab61860.1		p0712e02.24 [oryza sativa (japonica cultivar_group)] dbj bab89906.1 p0700a11.5 [oryza sativa (japonica cultivar_group)]	35,1	35_4008
Contig3892_at	np_193037.1		putative protein; protein id: at4g13010.1 supported by cdna: 6276. supported by cdna: gi_15028000 supported by	26,7	35_1404
HV05O23u_at	hv05o23u_at		no annotation	20.2.1	35_14861
Contig21251_at	t10255		nitrite transport protein chloroplast _ cucumber	34,13	35_7556
HV_CEa0014A18r2_at	aag01554.1	HvHsfB2c	heat shock transcription factor B2c	27.3.23	35_18576
Contig2014_at	t05739		probable heat shock protein 17 _ barley emb caa45861.1 (x64560) 17 kd heat shock protein [hordeum vulgare subsp. vul	20.2.1	35_14867
Contig9737_s_at	aal79714.1		putative senescence_associated protein [oryza sativa]	20.2.3	35_3925
Contig3198_at	contig3198_at		no annotation	35,2	35_15390
Contig18323_at	aag13582.1		(ac037425) unknown protein [oryza sativa]	35,2	35_8063
HVSMEn0016H11r2_s_at	hvsmen0016h11r2_s_at		no annotation	35,2	35_8063
Contig16980_at	np_567058.1		auxin_regulated protein; protein id: at3g57810.1 supported by cdna: 101256. supported by cdna: 29384. [arabidopsis	29.5.3	35_22714
Contig3761_at	aak26822.1		(af338432) starch branching enzyme iia variant [triticum aestivum]	2.1.2.3	35_15640
HF21J01r_at	np_174269.1		(nm_102716) hypothetical protein; protein id: at1g29760.1 [arabidopsis thaliana]	35,2	35_13219
HS06C20u_at	np_192861.1		putative protein; protein id: at4g11220.1 supported by cdna: 23536. supported by cdna: gi_14334419 supported by	33,99	35_1261
HS06C20u_s_at	np_192861.1		putative protein; protein id: at4g11220.1 supported by cdna: 23536. supported by cdna: gi_14334419 supported by	33,99	35_1261
Contig6802_at	bab90152.1		calcyclin binding protein_like [oryza sativa (japonica cultivar_group)]	35,2	35_16153
Contig1381_at	aaf68995.1		metallothionein [porteresia coarctata]	35,2	35_14134
Contig13656_at	contig13656_at		no annotation	35,2	35_4515
Contig15180_at	np_174785.1		atp_dependent rna helicase putative; protein id: at1g35530.1 [arabidopsis thaliana]	28,1	35_21460
Contig20753_at	bab92552.1		putative diacylglycerol kinase [oryza sativa (japonica cultivar_group)]	11,3	35_10001
Contig2008_s_at	caa69172.1	HvHsp16.88-CI	17 kda class i small heat shock protein [hordeum vulgare subsp. vulgare]	20.2.1	35_14863
HB18H23r_s_at	hb18h23r_s_at	HvHsp17.5-CI	heat shock protein 17.5	20.2.1	35_14859
Contig2006_s_at	s72544	HvHsp17.7-CI	heat shock protein 17.9 _ pearl millet emb caa63903.1 heat shock protein 17.9 [pennisetum glaucum]	20.2.1	35_14861
Contig2007_s_at	caa69172.1	HvHsp16.88-CI	17 kda class i small heat shock protein [hordeum vulgare subsp. vulgare]	20.2.1	35_14869
Contig2012_s_at	s72544	HvHsp17.7-CI	heat shock protein 17.9 _ pearl millet emb caa63903.1 heat shock protein 17.9 [pennisetum glaucum]	20.2.1	35_14866
HY03K23u_at	s72544	HvHsp17.7-CI	heat shock protein 17.9 _ pearl millet emb caa63903.1 heat shock protein 17.9 [pennisetum glaucum]	20.2.1	35_14866
HY10H17u_at	hy10h17u_at		no annotation	35,2	35_47455
Contig26505_at	bac20874.1		(ap005296) putative fiber protein [oryza sativa (japonica cultivar_group)]	35,2	35_21339
Contig10284_at	np_191190.2		glycosyl hydrolase family 27 (alpha_galactosidase/melibiase); protein id: at3g56310.1 supported by cdna: gi_18377734	3.8.2	35_18279
Contig3811_at	t07610		wsi76 protein _ rice dbj baa05538.1 wsi76 protein induced by water stress [oryza sativa]	3.1.1.1	35_1095
Contig9310_at	np_191720.1		(nm_116026) putative protein; protein id: at3g61610.1 [arabidopsis thaliana]	3,5	35_17271
